# An eco-evo-devo genetic network model of stress response

**DOI:** 10.1093/hr/uhac135

**Published:** 2022-06-07

**Authors:** Li Feng, Tianyu Dong, Peng Jiang, Zhenyu Yang, Ang Dong, Shang-Qian Xie, Christopher H Griffin, Rongling Wu

**Affiliations:** Center for Computational Biology, College of Biological Sciences and Technology, Beijing Forestry University, Beijing 100083, China; Center for Computational Biology, College of Biological Sciences and Technology, Beijing Forestry University, Beijing 100083, China; Center for Computational Biology, College of Biological Sciences and Technology, Beijing Forestry University, Beijing 100083, China; Center for Computational Biology, College of Biological Sciences and Technology, Beijing Forestry University, Beijing 100083, China; Center for Computational Biology, College of Biological Sciences and Technology, Beijing Forestry University, Beijing 100083, China; Key Laboratory of Ministry of Education for Genetics and Germplasm Innovation of Tropical Special Trees and Ornamental Plants, College of Forestry, Hainan University, Haikou 570228, China; Applied Research Laboratory, The Pennsylvania State University, University Park, PA 16802, USA; Center for Computational Biology, College of Biological Sciences and Technology, Beijing Forestry University, Beijing 100083, China; Center for Statistical Genetics, Departments of Public Health Sciences and Statistics, The Pennsylvania State University, Hershey, PA 17033, USA

## Abstract

The capacity of plants to resist abiotic stresses is of great importance to agricultural, ecological and environmental sustainability, but little is known about its genetic underpinnings. Existing genetic tools can identify individual genetic variants mediating biochemical, physiological, and cellular defenses, but fail to chart an overall genetic atlas behind stress resistance. We view stress response as an eco-evo-devo process by which plants adaptively respond to stress through complex interactions of developmental canalization, phenotypic plasticity, and phenotypic integration. As such, we define and quantify stress response as the developmental change of adaptive traits from stress-free to stress-exposed environments. We integrate composite functional mapping and evolutionary game theory to reconstruct omnigenic, information-flow interaction networks for stress response. Using desert-adapted Euphrates poplar as an example, we infer salt resistance-related genome-wide interactome networks and trace the roadmap of how each SNP acts and interacts with any other possible SNPs to mediate salt resistance. We characterize the previously unknown regulatory mechanisms driving trait variation; i.e. the significance of a SNP may be due to the promotion of positive regulators, whereas the insignificance of a SNP may result from the inhibition of negative regulators. The regulator-regulatee interactions detected are not only experimentally validated by two complementary experiments, but also biologically interpreted by their encoded protein–protein interactions. Our eco-evo-devo model of genetic interactome networks provides an approach to interrogate the genetic architecture of stress response and informs precise gene editing for improving plants’ capacity to live in stress environments.

## Introduction

Stresses, such as soil salination and drought, are one of the most significant land crises that threaten global ecosystems and the productivity use of natural resources [7, 11]. One environmentally safe strategy for remediating stressed lands is afforestation with stress-adapted plants [3, 4, 79]. The selection and breeding of superior varieties that can grow in saline and drought soils critically rely upon a profound understanding of how stress resistance is genetically controlled. Several studies have begun to characterize the genetic basis of phenotypic adaptation to salt stress, suggesting that saline-adaptive traits are polygenic [8, 62, 76]. However, traditional forward genetic approaches are limited in charting a complete picture of the genetic architecture underlying stress resistance and tolerance because of the lack of genetic mutations in adaptive phenotypes [50]. Stimulated by next-generation sequencing and genotyping techniques, high-density genetic mapping and genome-wide association studies (GWAS) have emerged as powerful reverse genetic approaches for systematically identifying and mapping quantitative trait loci (QTLs) that affect stress resistance and adaptation [12, 18, 30, 40, 66].

Traditional reverse genetic strategies for mapping stress-adapted traits have two major drawbacks. First, current GWAS attempt to map individual environment-sensitive biochemical and physiological traits of stress-adapted species expressed in single environments, but fail to quantify and dissect how plants balance stress defense and growth performance, which forms a unified strategy allowing plants to respond to stressed environments [83]. At present, a quantitative, evolutionarily meaningful definition of stress resistance is lacking. Given its dynamic and environment-induced features, stress response can be better understood as an ecological evolutionary developmental (eco-evo-devo) process [20] by which a species becomes stress-adaptive by sensing stress-related environmental cues early in life and using this information to develop adaptive phenotypes in later stages of life [49]. This process experiences complex interactions among developmental canalization, phenotypic plasticity, and phenotypic integration. Developmental canalization (or robustness) represents the property of a specific genotype to produce a consistent phenotype from one environment (e.g. stress free) to the next (e.g. stress exposed) [63]. In contrast, phenotypic plasticity describes a genotype’s ability to produce different phenotypes in response to a stress [10, 19, 45, 71]. The plastic response of a genotype to environmental change is the consequence of phenotypic integration, i.e. multi-trait correlations due to genetic, developmental, and functional connections [46, 53]. These three properties may not be consistent, e.g. developmental canalization acts at a cost of phenotypic plasticity and vice versa, but they operate in a way that counteracts each other to ultimately produce variable or stable phenotypes over stress-induced environmental change. In this sense, any stress response and resistance can be quantified as the difference between trait development trajectories expressed in stress-free and stress-exposed environments [83]. Mapping stress response is equivalent to mapping such development-dependent trait differences using Sang *et al*.’s [51] composite functional mapping (coFunMap). Compared to functional mapping of complex traits expressed in a single environment [35, 75], coFunMap demonstrates increased power for QTL detection.

Second, existing GWAS analyze genotype–phenotype relationships at individual markers once at a time, without considering a global landscape of the genetic architecture underlying complex traits that are thought to involve all genes the organism may possibly carry throughout its genome [9]. The justification of whether this so-called omnigenic theory holds for stress response can be made by orchestrating all GWAS genes into a mathematical graph [41], with nodes coded by genetic effects of individual genes and edges coded by genetic interactions or epistasis between individual gene pairs. More recently, Wu & Jiang [74] integrated evolutionary game theory [14, 57] and predator–prey theory [2, 6] to develop a statistical model for reconstructing large-scale networks in big dataset. The salient property of this model is that it can disentangle a maximally informative interaction architecture; for example, while traditional networks can only capture the strength of an interaction but not its direction [60] or the direction of interaction but not its strength [22], this model can fully characterize the bidirectionality, sign and weight of interactions. Wu and Jiang further integrated developmental modularity theory [17, 37, 64] through high-dimensional statistical models to infer multilayer and multiplex interactome networks from any high dimension of data. Traditional strategies for interaction detection require a huge sample size that may not be met in a majority of practical studies, but Wu and Jiang’s model is much less reliant on sample size to reconstruct interaction networks.

Network reconstruction is based on the data measured on each sample, but in GWAS, the genetic effect of a SNP is a population concept, rather than one that is reflected by individual samples, making it difficult to infer SNP-SNP interaction networks. This issue has been overcome by implementing functional mapping to estimate genetic effects as a time-varying curve. Thus, by treating time points as individual samples, genotype–phenotype data in GWAS are converted into a data format of genetic effects appropriate for reconstructing networks [59]. Based on time-varying genetic effect curves, Wang *et al.* [68] reconstruct one of the first omnigenic interactome networks that can be used to study genotype-environment interactions for growth traits.

In this article, we integrate eco-evo-devo, coFunMap, and Wu and Jiang’s network model to formulate a unified framework for reconstructing omnigenic multilayer networks that mediate stress response. To validate the biological relevance of this framework, we analyze genetic data from two complementary (mapping and association) populations of Euphrates poplar (*Populus euphratica* Oliver). As the only tree species that can grow and reproduce in aridic deserts, Euphrates poplar has strong salt resistance [13, 21] and can be vegetatively propagated [1]. These characteristics, along with its high ecological value [39] and sequencing information [36, 47, 81], make Euphrates poplar a woody plant model system for genetic studies of desert adaptation. One important feature of soil desertification faced by Euphrates poplar is soil salinization resulting from the accumulation of soluble salts in the arable land layers [24, 52]. We mimic the desert environment of this species by conducting a salt-stress treatment containing a salt concentration similar to that in natural desert habitats. One of the first responses of plants to salinity is a decreased rate of root and shoot growth [72], with a reduction in root and shoot length and mass [54]. The difference of shoot and root growth traits in the saline condition from those in the salt-free condition results from interactions among developmental canalization, phenotypic plasticity, and phenotypic integration, which can be used to assess salt resistance, a type of stress response. Mapping and association populations were grown under salt-free and salt-exposed treatments using clonal replicates. Wang et al. [68] developed a genome-wide by environment interaction association (GWEIS) model and used it to reconstruct interactome networks for saline response-related traits for these two populations. Results from the eco-evo-devo model and GWEIS model provide complementary, previously uncharacterized insight into the genetic architecture of salt resistance.

**Figure 1:**
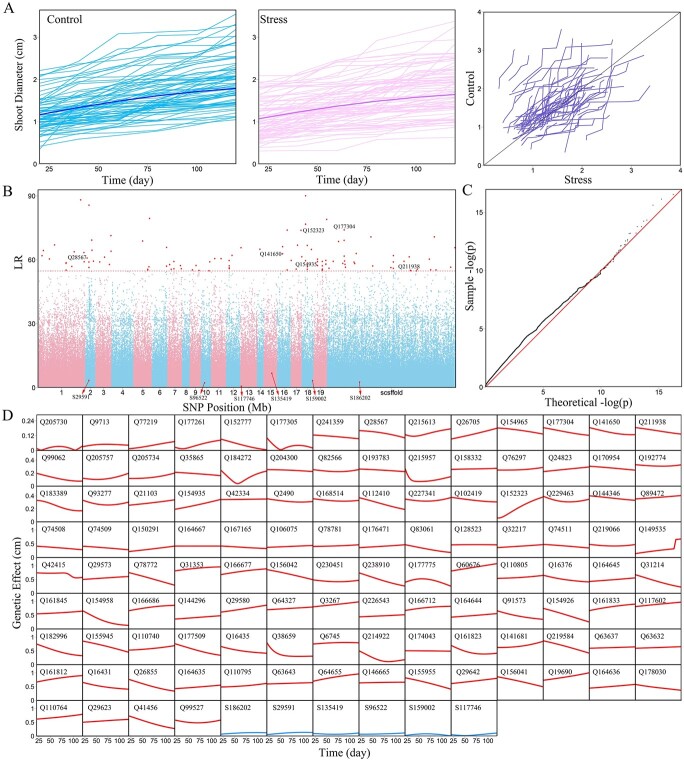
Identification of QTLs for the phenotypic plasticity of shoot growth trajectory in the GWAS population of Euphrates poplar. (**A**) Growth trajectories of shoots (thin lines) grown in salt-free (control) and salt-exposed tubes (stress) during the early ontogeny of juvenile trees (left two panels). The mean curve of the population is fitted by a logistic growth equation (1). Shoot growth curves are compared for the same genotype grown in control and stress conditions (rightmost panel). (**B**) Manhattan plot of log-likelihood ratios (LR) for phenotypic plasticity against different chromosomes estimated by coFunMap. Horizontal lines represent the critical thresholds of testcross markers (red line) and intercross markers (blue line) determined from 1000 permutation tests. Many QTLs detected reside at the genomic region of candidate genes (Table S1). (**C**) Q-Q plots of QTL detection by coFunMap. (**D**) Genetic effect curves of 116 QTLs on the phenotypic plasticity of shoot growth.

**Figure 2:**
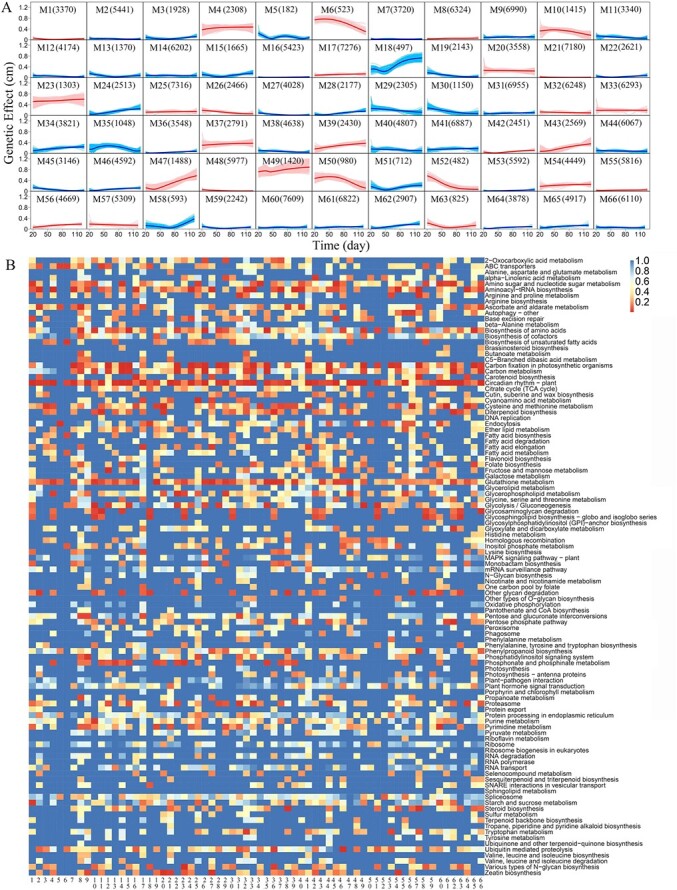
Functional clustering of genetic effect curves for the phenotypic plasticity of shoot growth trajectories in the GWAS population of Euphrates poplar. (**A**) Different patterns of genetic effect curves for 66 modules identified from 272 719 SNPs. Grey lines are effect curves of individual SNPs within modules, whose mean curve is indicated by a thick curve. QTL-containing modules are shown in red color. (**B**) The heatmap of gene function for 66 modules by KEGG analysis.

## Results

### Identification of stress response QTLs via GWAS

Shoot growth in the GWAS population follows an S-shaped curve, fitted by a logistic growth equation (Fig. 1A). In general, shoots display greater growth in control than stress treatment, with the size of difference proportional to salt tolerance or resistance of Euphrates poplar. We apply coFunMap to map treatment-dependent differences, which identifies 116 significant SNPs (named QTLs) for shoot growth-related salt resistance (Fig. 1B, C). GO analysis suggests that most of these QTLs can be annotated to candidate genes that mediate specific biological processes (Table S1). We further estimate the curves of genetic effects exerted by each QTL on salt resistance (Fig. 1D). Genetic effects increase with time for some QTLs but decrease with time for other QTLs. Some QTLs cyclically change their effects with time. Next, we will reconstruct multilayer interactome networks to investigate the genetic causes of why the QTLs tested by the conventional approach are significant and why insignificant SNPs are not significant. Answers to these questions can help design a better breeding scheme for utilizing salt resistant QTLs and for recovering the missing heritability of this trait.

### Networks of network

Network theory suggests that a big network would be automatically split or collapse into smaller subnetworks (i.e. modules) before it reaches a certain high dimension (Dunbar, 1992). Modules can be detected by clustering all SNPs into distinct groups according to their time-varying genetic effect patterns. SNPs within a group, i.e. module, form a subnetwork, which is linked to some extent with other subnetworks. We classify 272 719 SNPs into 66 modules using BIC minimization (Fig. 2A). Each module contains different numbers of SNPs (ranging from 182 to 7609) and displays different temporal patterns of genetic effects. One hundred and ten (110) QTLs detected by coFunMap are not evenly distributed among modules, rather are sporadically distributed in 27 of 66 modules. The number of QTLs contained in a module dramatically varies, ranging from 1 to 17. KEGG-based gene enrichment analysis shows that while some subnetworks share a similar set of biological functions, most networks perform a diverse range of biological functions (Fig. 2B). For example, both M32 (composed of 6248 SNPs) and M54 (composed of 4449 SNPs) encode aminoacyl-tRNA biosynthesis, carbon fixation in photosynthetic organisms, carbon fixation, and plant circadian rhythm, but the former specifically encodes tyrosine metabolism, and protein processing in endoplasmic reticulum, whereas the latter specifically encodes glutathione metabolism, aminoacyl-tRNA biosynthesis, zeatin biosynthesis, and steroid biosynthesis. Plant circadian rhythm is mediated by a majority of the modules. Taken together, 66 modules each form a relatively independent network community in a 272 719-node large-scale network for salt resistance, but they may also share some common biological functions.

We further divide each module into its distinct submodules, each composed of a reduced number of SNPs. Then we divide each submodule from every module into its distinct sub-submodules, each with the number of SNPs further reduced. This dendritic clustering procedure is repeated until the number of SNPs contained within a unit reaches a manipulatable size determined by Dunbar’s law. Through KEGG analysis, the overall biological function of each module can be explained by different submodules. Similarly, the overall biological function of each submodule is explained by different sub-submodules. At the end, we elucidate a coarse-grained and fine-grained classification taxonomy for the genetic network of salt resistance.

### Multilayer networks as a roadmap of QTL effects

**Figure 3:**
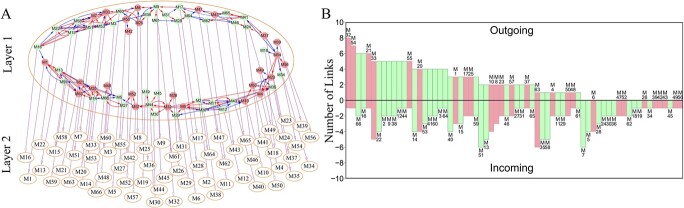
Multilayer interactome network in the GWAS population of Euphrates poplar. (**A**) The top layer genetic network reconstructed from 66 modules, each forming a second-layer network. Arrowed red and blue lines stand for activation and inhibition, respectively, with the thickness of lines proportional to the strength of regulation. (**B**) Distribution of the number of outgoing and incoming links across modules. QTL-containing modules are shown in red color.

After all SNPs are clustered into hierarchical structure (smaller units contained within larger), we reconstruct genetic networks among modules, submodules, sub-submodules, and so on to form a multilayer interactome graph. At the top (first-layer) of the graph is a 66-node inter-module network (Fig. 3A). Nodes in the top-layer network each represent the mean effects on salt resistance of all SNPs from a module and its edges represent interactions between the mean effects of different modules. At the second layer, there are 66 inter-submodule interaction networks reconstructed from the mean genetic effects of all SNP within submodules. Each submodule has multiple inter-sub-submodule interaction networks reconstructed from the mean genetic effects of all SNP within sub-submodules. At the bottom, genetic networks are constructed by the genetic effects of individual SNPs, which are distributed like patches and smaller patches within larger patches in ecosystems [33].

Multilayer interactome networks provide a detailed framework to dissect salt resistance at different levels of resolution from collective to individual effects. Links in a network can be outgoing if a node affects a second node or incoming if a node is affected by a second node. While outgoing links are associated with leaders, incoming links are associated with workers. Thus, the relative number of outgoing and incoming links can be used to evaluate the position of a node. In the first-layer network, we identify modules M32, M54, M66, M16, and M21 as primary leaders not only with many more outgoing links than average, but also with a minimum number of incoming links (Fig. 3B), which play a pivotal role in maintaining network structure and organization. About 30% of modules only have incoming links, rather than exert outgoing links, suggesting that these modules serve workers. We find that the number of QTLs within a module has no positive association with the number of outgoing links, but is slightly associated with the number of incoming links. Modules M32 and M54 each containing one QTL have 8 and 7 outgoing links, respectively, whereas M23 and M37 with 15 and 11 QTL, respectively, only have two outgoing links. Many QTL-rich modules have many incoming links; e.g. M6 with as many as 8 QTLs has more incoming links than average. Taken together, QTL modules tend to serve as workers that more directly participate in modulating the salt resistance of Euphrates poplar.

**Figure 4:**
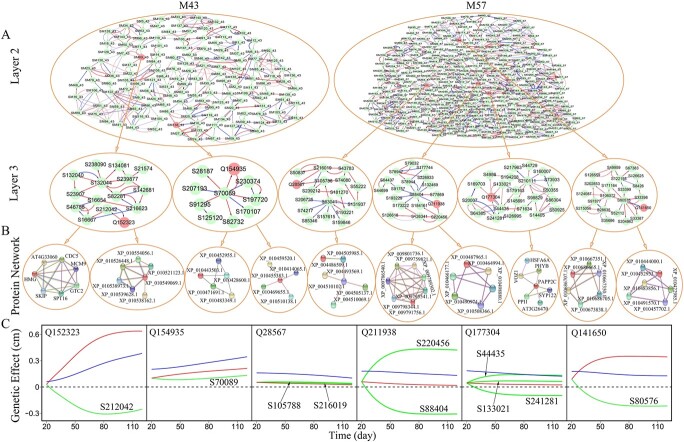
Multilayer genetic networks of QTL-containing modules M43 and M57 in the GWAS population of Euphrates poplar. (**A**) The second-layer network of submodules from each module and the third-layer network of SNPs from QTL-containing submodules. Arrowed red and blue lines stand for activation and inhibition, respectively, with the thickness of lines proportional to the strength of regulation. QTLs and QTL-containing submodules are shown in red. (**B**) Protein–protein interaction networks coded by SNP-SNP interactions detected from multilayer networks. (**C**) Genetic effect curves of QTLs including their net effect curves (blue), independent effect curves (red) and dependent effect curves (green) due to regulation by other SNPs (whose names are indicated).

We reconstruct the second-layer submodule networks for each of the 66 modules, in which individual SNP-based networks at the third layer are inferred. Modules M43 (containing 2 QTLs) and M57 (containing 4 QTLs) form 143- and 303-node submodule second-layer networks, respectively (upper panel, Fig. 4A). We further reconstruct the third-layer (bottom) networks at the SNP level for each sub-submodule from each module, illustrating the SNP networks of QTL-containing sub-submodules (lower panel, Fig. 4A). To verify if SNP-SNP interactions detected truly exist, we conduct a detailed pathway analysis to build protein–protein interaction networks based on annotated genes (Fig. 4B). In the SNP network of submodule SM69_43 of module M43, the expression of S134081 residing at gene LOC105113742 is promoted by S239877 in proximity to gene LOC105115805. These two genes encode mitogen-activated protein kinase 9 protein XP_010549069.1 and trihelix transcription factor GTL1-like protein XP_010538162.1, respectively, that are observed to interact with each other to mediate stress resistance. We infer that XP_010549069.1-XP_010538162.1 interaction may be encoded by S134081-S239877 interaction that is detected by the network model. In the SNP network of submodule SM163_57 of module M57, S44435 inhibits S64365 but the latter promotes the former. This aggressive/altruistic relationship can be justified by interactions between transcription factor VOZ1 protein (encoded by S44435) and PAPP2C (probable protein phosphatase 2C 9) protein (encoded by S64365) (Fig. 4B). Pathway analysis of all SNP networks suggests that, of all SNP-SNP interactions detected, as high as >80% can be interpreted by protein–protein interactions, implying the biological relevance of interaction detection by the model.

From the SNP networks, we can chart a complete roadmap of how a QTL affects salt resistance in Euphrates poplar. By decomposing the net genetic effect of a QTL into its independent and dependent effects (see the Methods), we can characterize how each QTL exerts its impact on the plastic response of shoot growth to salt stress. Q154935 in SM138_43 and Q28567 in SM11_57 each have a small independent effect; i.e. these QTLs do not affect salt resistance when they are in isolation (Fig. 4C). However, they are observed to have significant genetic effects because of the pronounced up-regulation they receive from other loci. For example, Q154935 with a subtle intrinsic effect is positively regulated by S70089, making it a significant QTL. To verify if such regulation exists, we conduct a pathway analysis. Q154935 and S70089 reside in genes LOC105134122 and LOC105131903, which encode chloroplastic APO protein 2 (XP_010483349.1) and protein disulfide-isomerase LQY1-like protein XP_010428600.1, respectively. These two proteins are found to mediate plants’ response to abiotic stress through their strong interaction [82] (Fig. 4B), which justifies the detection of Q154935-S70089 interaction. Q211938 in SM112_57 and Q177304 in SM163_57 are each simultaneously up-regulated and down-regulated by different loci, but because the overall regulation is positive, these QTLs still display significant net effects, despite their small independent effects (Fig. 4C).

**Figure 5:**
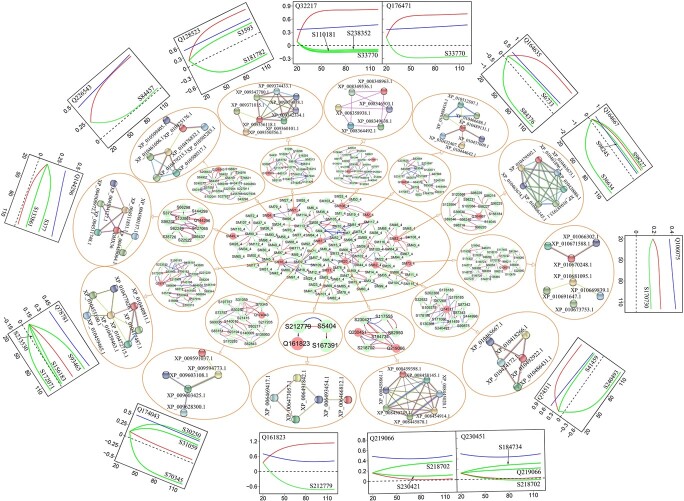
Multilayer genetic networks of QTL-containing module M4 in the GWAS population of Euphrates poplar. At the core is the second-layer network of 117 submodules (with QTL-containing submodules indicated in red). At the second circle from the core are the SNP-based networks of QTL-containing submodules, followed by protein–protein interaction networks coded by SNP-SNP interactions. Arrowed red and blue lines stand for activation and inhibition, respectively, with the thickness of lines proportional to the strength of regulation. At the outer circle are the genetic effect curves of individual QTLs, including their net effect curves (blue), independent effect curves (red) and dependent effect curves (green) due to regulation by other SNPs (whose names are indicated).

Different from the above-mentioned QTLs, the observed effects of Q152323 in SM69_43 and Q141650 in SM249_57, though significant, are lower than their independent effects because they are substantially down-regulated by other loci. For example, in the SNP network of submodule SM69_43, Q154935 (located at gene LOC105111422) is negatively regulated by regulator S212042 (located at gene LOC105115006). The existence of such an interaction between Q154935 and S212042 is verified by pathway analysis, which suggests that LOC105111422 encodes FACT complex subunit SSRP1 protein HMG that interacts with peptidyl-prolyl cis-trans isomerase CYP57 protein AT4G33060 encoded by LOC105115006 to determine plant response to stress [56] (Fig. 4B). We reconstruct multilayer interaction networks for QTL-rich module M4, in which the SNP networks of 12 QTL- containing submodules allow us to dissect how each QTL interacts with other SNPs to mediate salt resistance (Fig. 5). Some QTLs, such as Q106075 in SM74_4, have a large independent effect, which is further up-regulated to produce even more substantial net genetic effects. Pathway analysis shows that SNP-SNP interactions (involving or not involving QTLs) can be interpreted via protein–protein interactions (Fig. 5). As illustrated, multilayer interaction networks provide an encyclopedia to document the roadmap of how each QTL affects salt resistance in Euphrates poplar.

### Detecting undetected genetic effects and retrieving missing heritability

**Figure 6:**
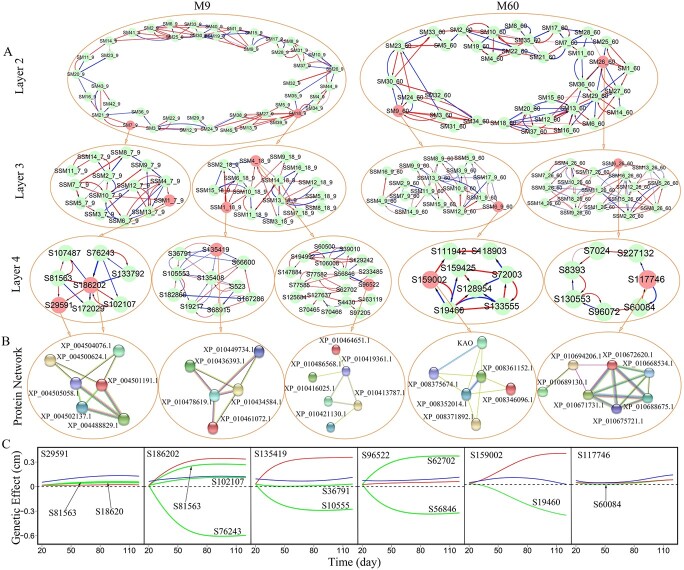
Multilayer genetic networks of QTL-absent modules M9 and M60 in the GWAS population of Euphrates poplar. (**A**) The second-layer network of submodules from each module and the third-layer network of SNPs from the submodules that contain chosen insignificant SNPs (indicated in red). Arrowed red and blue lines stand for activation and inhibition, respectively, with the thickness of lines proportional to the strength of regulation. (**B**) Protein–protein interaction networks coded by SNP-SNP interactions detected from multilayer networks. (**C**) Genetic effect curves of chosen insignificant SNPs including their net effect curves (blue), independent effect curves (red) and dependent effect curves (green) due to regulation by other SNPs (whose names are indicated).

From multilayer interactome networks, we can further reveal the underlying mechanisms of why a SNP is insignificant using coFunMap. We choose two QTL-absent modules M9 and M60 to reconstruct four-layer genetic networks (Fig. 6A). As can be seen from a pathway analysis, in SNP networks of sub-submodules at the fourth layer, SNP-SNP interactions can be interpreted by the coded protein–protein interactions (Fig. 6B). In the SNP network of sub-submodule SSM1_7_9 from SM7 of M9, S135419 (at gene LOC105128220) is insignificant for salt resistance by coFunMap, but virtually it has a marked independent effect which could be expressed if this SNP was negatively regulated by S105553 (at gene LOC105140270) (Fig. 6C). In other words, down-regulation by S105553 hides the independent expression of S135419.

Although S135419 also is up-regulated by SNP S36791 (at gene LOC105128220), this up-regulation is not large enough to compensate for the down-regulation of S105553. A pathway analysis suggests that S135419-S105553 and S135419-S36791 interactions detected in SNP networks can be well explained by the interactions of putative pre-mRNA-splicing factor ATP-dependent RNA helicase DHX16 protein (XP_010436393.1), coded by S135419, with eukaryotic translation initiation factor 4E-1 protein (XP_010434584.1), coded by S105553, and with DEAD-box ATP-dependent RNA helicase 5 protein (XP_010478619.1), coded by S36791, respectively (Fig. 6B).

In the SNP network of sub-submodule SSM4_18_9, S96522 is up-regulated by S62702, and simultaneously down-regulated by S56846 to a larger extent. The up-regulation and down-regulation are almost equal, making the net effect of S96522 smaller than its independent effect (Fig. 6C). The identification of protein–protein interactions by pathway analysis verifies the existence of interactions between S96522 and SNP62702 and S56846. Some SNPs are up-regulated and down-regulated by multiple SNPs, but the overall dependent genetic effect is negative because up-regulation is collectively larger than down-regulation (Fig. 6C). S29591 and S117746 are located in the SNP networks of sub-submodules SSM1_7_9 and SSM6_9_60, respectively, both of which are so small in both independent and dependent effects that their net genetic effects are not amplified to an observable level (Fig. 6C). All these SNP-SNP interactions described above can be annotated to protein–protein interactions.

Through a detailed effect component analysis, we find that all insignificant SNPs can be broadly classified into three categories. In the first category, SNPs have large intrinsic capacity for independent genetic effects, but they are not significantly expressed because of strong negative regulation from other loci. The second category includes those SNPs with small independent effects, which receive both strong positive and negative regulation from different loci. The cancellation of positive and negative regulation does not contribute to increase net genetic effects. In the third category, SNPs have small independent effects and do not receive any regulation or are slightly regulated in either positive or negative direction by other loci, in which case no genetic effects can be observed. For the first two categories, insignificant SNPs can be made to be significant for salt resistance by inhibiting the genetic expression of negative regulators and/or promoting the genetic expression of positive regulators through genetic manipulations. Thus, missing heritability hidden within these types of SNPs can be recovered and utilized for practical genetics. Taken together, reconstructing fine-grained SNP genetic networks at the SNP level enables the reconstruction of the roadmap describing how any genetic locus affects salt resistance, through direct or indirect pathways.

### Mapping validation of salt-resistant multilayer networks

To validate the biological relevance of the multilayer networks used to dissect salt resistance for Euphrates poplar, we conduct a complementary experiment using a mapping population of the same species. By culturing shoot cuttings of a full-sib family derived from two wild trees in salt-free and sale-exposed media, we measure and dissect salt resistance expressed as the difference of total adventitious root growth between two conditions (Fig. S1A). We implement coFunMap to identify 27 QTLs for root growth-related salt resistance on a high-density linkage map constructed from 8305 SNPs covering the 19 *Populus* chromosomes (Fig. S1B). GO analysis suggests that most of these QTLs can be annotated to candidate genes that mediate specific biological processes (Table S2). We further estimate the curves of genetic effects exerted by each QTL on salt resistance, from which the pattern of how QTLs change their genetic effects with time can be characterized (Fig. S1C). We implement functional clustering to classify 8305 SNPs into 403 modules based on BIC (Fig. S2A), thus forming 403 network communities, of which 15 contain 1–6 QTLs. Tremendous variability in time-varying genetic effects implies a complex network of module-module interactions and interdependence (Fig. S2B). We further cluster each module into its distinct submodules and cluster submodules into sub-submodules if the number of SNPs within these submodules is greater than Dunbar’s number.

**Figure 7:**
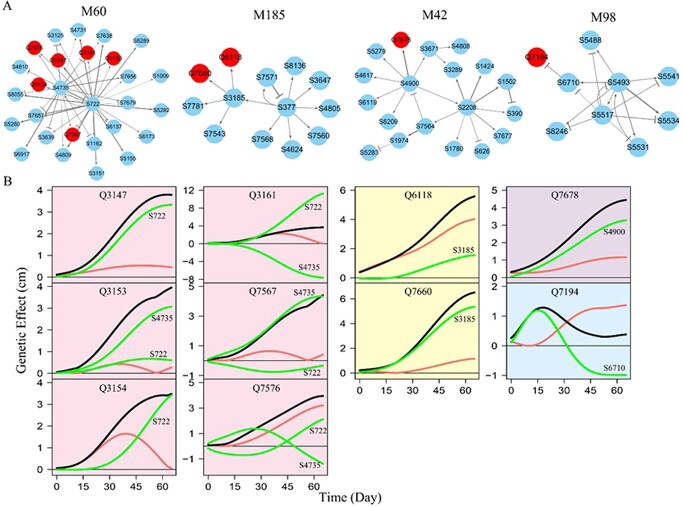
Fine-grained genetic networks at the SNP level for QTL-containing modules underlying the phenotypic plasticity of root growth trajectories in the mapping population of Euphrates poplar. (**A**) The SNP networks of modules M60, M185, M42, and M98, where arrowed lines and T-shaped lines stand for activation and inhibition, respectively, with the thickness of lines proportional to the strength of regulation. QTLs are highlighted in red. (**B**) Net genetic effect curves (black line) of each QTL from the above four different SNP networks are decomposed into independent effect curves (red line) and dependent effect curves (green line) due to regulation by other SNPs.

We reconstruct genetic networks among modules, submodules, sub-submodules, and SNPs, forming a multilayer interactome network for salt resistance in terms of how root growth responds to the saline condition. At the first layer of the multilayer network is the 403-node inter- module network in which 15 QTL-containing modules tend to serve as workers because they have higher more incoming link counts. Each module in the first-layer network decomposes into multiple second-layer networks, each of which further decomposes into the third-layer networks or SNP networks (Fig. 7A; Fig. S3). The SNP networks allow us to decompose the net genetic effect of a QTL into its independent effect and dependent effect from which we can characterize how each QTL affects the plastic response of root growth to salt stress. Q3147 and Q3153 in M60 and QTL 7567 in M185 each have very small independent effects, but they display significant genetic effects in our observations. This is because these QTLs receive significant up-regulation from regulators (Fig. 7B). It is interesting to find that Q3161 in M60 is substantially up-regulated by S722 and also, similarly, down-regulated by S4735, ultimately producing a net genetic effect slightly different from the independent effect because of the cancellation of up- and down-regulation. Yet, such up- and down-regulation activates Q3161 to produce about 30% of genetic variation in the plastic response of adventitious root growth to salt stress. Q6118 in M185 has a large independent effect, and this effect is augmented through up-regulation from S3185 to produce an even larger net genetic effect (Fig. 7B). We dissect the underlying mechanisms of the significance of all other QTLs for root growth-related salt resistance in Euphrates poplar (Fig. S3), gaining unique insights into the genetic architecture of salt resistance supporting the findings from the GWAS experiment.

**Figure 8:**
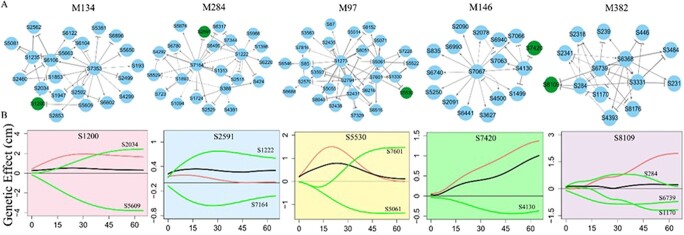
Fine-grained genetic networks at the SNP level for QTL-absent modules underlying the phenotypic plasticity of root growth trajectories in the mapping population of Euphrates poplar. (**A**) The SNP networks of modules M134, M284, M97, M146, and M382, where arrowed lines and T-shaped lines stand for activation and inhibition, respectively, with the thickness of lines proportional to the strength of regulation. (**B**) The SNPs chosen for a further analysis are highlighted in green. Lower: Net genetic effect curves (black line) of each SNP from the above five different modules are decomposed into independent effect curves (red line) and dependent effect curves (green line) due to regulation by other SNPs.

We randomly choose five insignificant SNPs at the bottom of the Manhattan plot (Fig. S1B), which are shown to have small observed genetic effects (Fig. S1C). These insignificant SNPs are from different modules. In the SNP network of module M134 (Fig. 8A), S1200 is observed to have a small genetic effect on salt resistance, but it has a sizeable independent effect which could be expressed if this SNP were not regulated by other loci (Fig. 8B). S1200 is up-regulated by S2034, but is, at the same time, down-regulated by SNP5609 to a larger extent. The interaction of the up-regulation and down-regulation makes the net effect of S1200 smaller than its independent effect. In practice, the potential impact of S1200 on salt resistance can be utilized by blocking the down-regulation of S5609. As pointed out from the GWAS experiment, this strategy may provide a means of retrieving missing heritability.

In the SNP network of module M284, both the observed effect and independent effect of S2591 are small (Fig. 8B). However, this SNP can still be used if the down-regulation it receives from S7164 is blocked, with the up-regulation by S1222 preserved. Based on the pattern of up-regulation and down-regulation, we can interpret the effects of other three insignificant SNPs from modules M97, M146, and M382 (Fig. 8B). The biological relevance of SNP-SNP interactions can be interpreted by GO analysis (Fig. S4). From SNP genetic networks, we can illustrate the precise genetic pathways for each genetic locus to mediate salt resistance.

## Discussion

The degree at which a plant reduces its growth from a salt-free to salt-stress environment reflects the plant’s capacity to defend against stress. We conceptualize stress response as an eco-evo-devo process involving complex interactions among developmental canalization, phenotypic plasticity, and phenotypic integration. Because of this conceptualization, we can measure and use the change of growth traits from non-stressed to stressed environments as a quantitative trait of stress response. This, in combination with functional mapping and evolutionary game theory, enables us to reconstruct omnigenic, multilayer interactome networks for stress response. Beyond traditional genetic approaches that can only identify individual stress genes or pathways [33], such eco-evo-devo networks can chart a more complete atlas of genetic control over stress tolerance and resistance. We test and validate such multilayer interactome stress networks using two complementary ecological genetic experiments of desert-adapted Euphrates poplar.

We first use coFunMap to characterize significant QTLs for shoot and root growth-related salt resistance. Many of these QTLs are detected to be significant not because of their intrinsic capacity to exert a genetic effect but rather because they are promoted by regulators. For example, Q3147 on chromosome 5 (from module M60; Fig. 7) detected from the mapping experiment is proximal to *gene RPL38A* encoding a structural constituent of ribosomes, whose marked effect on salt resistance is due to strong up-regulation by S722 (at LOC105121577 encoding protein transport) as a positive regulator (Fig. S4). This result suggests that, to precisely implement Q3147 into the improvement of salt resistance, marker-assisted selection or gene editing should not only focus on this locus alone, but rather on this locus and its regulator S722 as a cohesive unit. Q5065 on chromosome 10 (from module M260; Fig. S3) is observed to be significant for its net effect, but its independent effect is even larger. A negative regulation by S7221 (residing at LOC1051413581, encoding the lysine biosynthetic process via diaminopimelate) inhibits the expression of Q5065’ independent effect (Fig. S4). Thus, to maximize the effect of this QTL on salt resistance, a knock-off approach is needed to block the regulation of S7221.

The second benefit of our multilayer interactome networks is in retrieving missing heritability. Although deep sequencing techniques have been used, missing heritability, i.e. undetected genetic variation, is still a common phenomenon for complex trait or disease mapping in current GWAS [42]. Tremendous efforts have been made to excavate hidden genetic variation by identifying more genetic variants or adding new non-genetic variants [16, 34, 44, 77], but there is little literature on retrieving missing heritability from a methodological perspective. While phenotypic plasticity has been widely recognized as a complex trait [29, 43, 55], a stress-induced form of phenotypic plasticity - salt resistance is likely to be under omnigenic control [9]. Thus, our multilayer interactome network that cover a complete set of genome-wide SNPs and their interactions affords a unique opportunity to detect cryptic genetic variation that is undetected by conventional analysis approaches. In our genetic experiments on Euphrates poplar, we found numerous examples in which a SNP detected to be insignificant by coFunMap displays a strong independent genetic effect on salt resistance that is masked by net down-regulation from other loci. For example, insignificant S1200 on chromosome 2 in the mapping experiment has a fairly strong independent effect (Fig. 8); i.e. it may affect salt resistance, to a large extent, on its merit if its incoming negative down-regulation from S5609, located within gene *UBA1* encoding cellular response to DNA damage stimulus (Fig. S4), is silenced. In the GWAS experiment, insignificant S2591 on chromosome 4 has no strong independent effect (Fig. 6), but it is simultaneously promoted by S1222 and inhibited by S7164, with up-regulation and down-regulation canceled out to form subtle net genetic effects. Thus, if the negative regulation from S7164 is blocked, S2591 could exert a stronger net genetic effect on salt resistance as a result of positive up-regulation from S1222. In short, multilayer interactome networks enable detection of genetic variation hidden by negative gene co-regulation, allowing potential use of these variations to improve salt resistance.

Two independent ecological genetic experiments of Euphrates poplar provide complementary results that reciprocally justify the power of multilayer interactome networks. For example, the mechanisms by which a QTL’s net effect on the salt resistance of shoot growth is due to its intrinsic capacity or positive promotion by other regulators in the GWAS experiment are also detected for the QTLs associated with the salt resistance of root growth in the follow-up experiment. To confirm the biological relevance of SNP-SNP interactions detected from multilayer interactome networks, we performed extensive protein-based pathway analysis for each interaction. If two SNPs each are close to a different gene encoding a certain protein of specific molecular function, then interactions of these two SNPs through promotion or inhibition would inevitably lead to links between their encoding proteins. In the SNP network of SSM4_18_9 in the GWAS experiment (Fig. 6), S96522, S56846, and S62702 are annotated to genes LOC105137146, LOC105123926, and LOC105107215 that encodes IQ-DOMAIN 1-like protein (XP_010419361.1), probable prefoldin subunit protein (5XP_010421130.1), and phosphatidylinositol 4-phosphate 5-kinase 9-like protein (XP_010464651.1), respectively. It is likely that both strong up-regulation of S62702 and strong down-regulation of S56846 for S96522 lead to tight XP_010419361.1 to 5XP_010421130.1 and XP_010419361.1 to XP_010464651.1 links, as detected by pathway analysis (Fig. 6). A systematic pathway analysis shows that a majority of SNP-SNP interactions (> 80%) detected from multilayer interactome networks can precisely map to protein–protein interactions, demonstrating their biological interpretability.

Despite its ecological, environmental, and social values, Euphrates poplar is still an underrepresented species in modern genetic studies. However, its strong adaptation to saline environments offers value in the mechanistic exploration of desert adaptation and, more importantly, tips for how to best characterize the genetic architecture of complex traits in general. Both mapping and GWAS experiments using this hardy species characterize consistent, yet previously undetected genetic mechanisms that can be better used in ecological protection and resistance breeding programs. As one of the first genetic network studies of salt resistance based on a common-garden experiment, our study can be expanded to more precisely reveal the genetic mechanisms of this important ecological process. First, we mimic the salination environment to which Euphrates poplar adapts based on desert soil salination. To better mimic the environmental extreme of saline soils, multifactorial stressors, including salinity, drought, and nutrient deficiency, etc., should be jointly considered. Second, our study is conducted using salt-adapted Euphrates poplar, but a phylogenetically close, salt-sensitive poplar should be included in the common-garden experiment to reveal genetic specificity for desert adaptation. Third, the study populations used have a modest sample size (*n*$\approx$ 100), from which the results should be interpreted with caution. However, information from our sample size is amplified by precise phenotyping (multiple clonal replicates under two levels of treatment) and repeated phenotyping (high-density time series measurement of phenotypes). As indicated by our previous simulation studies [84], such augmented information is sufficient to provide relatively accurate estimates of genetic effects by marginally-based conventional mapping approaches. Because our network reconstruction is based on predator–prey (generalized Lotka-Volterra), specified by ODEs [14, 69], built from the curves of genetic effects, rather than on sample size, estimates of gene–gene interactions in the multilayer networks are much less sample size-dependent.

Our eco-evo-devo network model was validated by analyzing two salt-resistance dataset of Euphrates poplar. The model contains two statistical cores: functional mapping and ODE solving. Statistical properties of functional mapping have been investigated by computer simulation in Wang et al. [68], suggesting that functional mapping can be reasonably used in practical genetic experiments with a moderate sample size and high phenotyping precision. Jiang et al. (2019) preformed computer simulation to examine the statistical behavior of interaction detection through ODEs under different numbers of time points and different measurement errors. From such simulation, we infer that the power and positive false rate of SNP interaction detection can be reasonably met with our well-controlled Euphrates poplar experiments.

The application of our model can be leveraged to any biological process related to the phenotypic plasticity of traits to environmental signals by treating it as a quantitative trait. This will expand our model into a broad domain of application to environmental adaptation. From an evolutionary perspective, the eco-evo-devo network model is alternative to Wang et al.’s [68] GWEIS network model. Both models share the similarity of methodological foundation on the integration of functional mapping, evolutionary game theory and predator–prey theory. However, the two models differ dramatically in many aspects of modeling gene–environment relationships. First, the eco-evo-devo model focuses on inferring the genetic network of phenotypic plasticity, whereas the GWEIS model on characterizing the environmental plasticity of genetic networks. Second, the eco-evo-devo model explores the genetic basis of how traits respond to environment, providing critical information for evolutionary geneticists to understand the evolution of phenotypic plasticity. On the other hand, the GWEIS model captures the environment-dependent change of how genes control the trait, which can help breeders select superior traits for specific environments. Third, the eco-evo-devo model can extract the role of genes in altering phenotypes, whereas the GWEIS model can excavate the environmental impact on genetic control. In practice, we recommend that these two models be used simultaneously in order to better disentangle the genetic architecture of phenotypic plasticity including stress response.

Thanks to the development of high-dimensional statistical theory and methods in the past decades [5, 61, 65, 67, 85], our network reconstruction has overcome the obstacle of high-dimensional marker data. The fundamental principle of inferring large-scale networks is derived from developmental modularity theory [17, 37, 64], suggesting that a complex system can be broken down into its distinct but interconnected modules (or network communities). Individual modules can be further broken down into distinct submodules, which each are broken down into distinct sub-submodules. This clustering process is repeated until the number of SNPs within a unit reaches a tractable number. Ultimately, our genetic network is a tridimensional, multilayer, multiplex, and sparse, at the top layer expressed as the coarse-grained inter-community network and at the bottom layer as fine-grained SNP-SNP networks. High-resolution SNP networks canalize a precise roadmap though which geneticists can browse and trace the spatiotemporal trajectories of how each gene directly or indirectly affects stress response.

## Materials and methods

### GWAS and mapping experiment of salt resistance

We apply two experimental populations of Euphrates poplar to validate our models. A detailed establishment procedure of these two populations were described in Wang et al. [68] and Zheng *et al.* (2017). One population includes a panel of wild trees sampled from the species’ natural distribution (GWAS), whereas the other is a full-sib family derived from two wild trees (genetic mapping). Thus, the two experiments are complementary in genetic dissection and can be used to validate the findings of each other. The GWAS population has 121 tree genotypes, genotyped by 6 978 931 SNPs, of which 272 719, passing the tests of Hardy–Weinberg equilibrium and minor allele-segregation, are used for the subsequent analysis. Using fastStructure software [48], we find that these 121 trees come from a single population. The mapping population contains 106 F_1_ hybrids, from which a high-density linkage map of length 4575 cM was reconstructed from 8305 SNPs [80].

Both populations have been clonally propagated, having the same genotype planted with three or more clonal replicates in salt-free medium (control) and salt-exposed medium (containing 0.1% NaCl) (stress). Shoot heights were measured for the GWAS population once every 20 days during the 120 days of culture, totaling six evenly-spaced measurement time points. The total lengths of all adventitious roots were measured for the mapping population, starting 13 days after the buds were cultured in the tubes, followed by repeated measurements at a frequency of once every 5 days until there was no space in the tubes for growth (day 78). The change in the phenotype of the same genotype from control to stress conditions reflects the capacity of this genotype to exert salt resistance.

### Composite functional mapping

Let **x***_i_* = (*x_i_*(*t*_1_), …, *x_i_*(*t_T_*)) and **y***_i_* = (*y_i_*(*t*_1_), …, *y_i_*(*t_T_*)) denote a vector of phenotypic values for a trait measured at time schedule (*t*_1_, …, *t_T_*) under control (X) and stress (Y) conditions for the *i*th individual from a population, respectively. The time-varying differences of trait values for the *i*th individual between two conditions are computed as.


(1) \begin{align*}& \textbf{z}_{i} = (z_{i}(t_{1}),..., z_{i}(t_{T})) \equiv ((xi(t_1) - y_i(t_i)),..., (x_i(t_T)- y_i(t_T))) \end{align*}

where the size and sign of each element at a specific time point reflect the degree and pattern of how individual *i* responds to salt stress over the time course. The **z** vector represents adaptive changes in stress response during ontogenetic development. We implement Sang *et al.*’s [51] coFunMap to map stress response traits and estimate the time-varying genetic standard deviation of a SNP, denoted by ${g}_s(t)$, *s* = 1, …, *m*, where *m* is the total number of SNPs from a population.

### Network reconstruction

Sun *et al.* [59] introduced functional mapping and evolutionary game theory [14, 57] to decompose the net genetic effect of a SNP into its independent effect and dependent effect component. This decomposition can be mathematically formulated by a set of ordinary differential equations (ODEs), i.e.

(2) \begin{align*}& \frac{d{g}_s(t)}{dt}={Q}_s\left({g}_s(t);{\Theta}_s\right)+\sum_{s^{\prime }=1,{s}^{\prime}\ne 1}^m{Q}_{s\leftarrow {s}^{\prime}}\left({g}_{s^{\prime }}(t);{\Theta}_{s\leftarrow {s}^{\prime }}\right), \end{align*}

where $d{g}_s(t)/ dt$ reflects the net genetic effect of SNP *s* on stress response at time *t*, ${Q}_s(\bullet )$ is a time-varying function that specifies the independent genetic effect of SNP *s* assuming that it is isolated, ${Q}_{s\leftarrow {s}{^\prime}}(\bullet )$ is a time-varying function that specifies the dependent genetic effect of SNP *s* that results from the impact of other SNP ${s{^\prime}}$ on it, and ${\Theta}_s$ and ${\Theta}_{s\leftarrow {s}{^\prime}}$ are a set of ODE parameters that fit the independent and dependent functions, respectively. We implement Legendre Orthogonal Polynomials to smooth the independent and dependent functions.

We incorporate LASSO-based variable selection [61, 67, 85] to select a small set of SNPs that are linked with a given SNP in the most significant way. The net genetic effect of a SNP is considered as a response, whereas those of all other SNPs are considered as predictors. Thus, a regression is built and used for variable selection. After model selection, the ODEs of equation (2), representing a full model in terms of its full interconnections, are reduced to a small set of the most significant SNPs for each SNP. The reduced ODEs can be solved numerically with the fourth-order Kunge-Kutta algorithm. The independent genetic effects of different SNPs are coded as nodes and the dependent genetic effects of SNP pairs coded as edges into mathematical graphs. This graph represents a maximally informative network filled with bidirectional, signed, and weighted interactions.

### Detecting network communities

When Equation (2) contains tens of thousands of dimensions, it cannot be readily solved. According to developmental modularity theory, we divide a large-scale network into multiple distinct network communities. Components within a network community are connected more closely with each other than those from other communities. Communities can be detected by using functional clustering [28, 70] to classify all *m* SNPs into different modules based on the similarity of the temporal pattern of their genetic effects. Each module represents a distinct network community. Through this clustering, we can ultimately reconstruct multiple interconnected smaller networks from any number of SNPs. Thus, the model can overcome the curse of network dimensionality.

## Acknowledgements

We thank members at Beijing Forestry University Center for Computational Biology for their contributions to data collection. This work is supported by Beijing Forestry University Research Fund.

## Author contributions

FL derived the model, analyzed the data, and wrote the code. TD, PJ, ZY, AD, and SX participated in data analysis. CG introduced game theory. RW conceived and supervised the study and wrote the manuscript within inputs from CG.

## Data and Code availability

All data and code are publicly available at https://github.com/CCBBeijing/PPMultilayerNetwork/tree/main/Euphrates and can also be requested from the corresponding author.

## Competing interests

The authors declare no competing financial interests.

## Supplementary data

Supplementary data is available at *Horticulture Research * online.

## Supplementary Material

Web_Material_uhac135
