# Supplementary material for: An eco-evo-devo genetic network model of stress response
Source: Hortic Res. 2022 Jun 7;9:uhac135. doi: 10.1093/hr/uhac135 (PMC9433980; doi:10.1093/hr/uhac135)

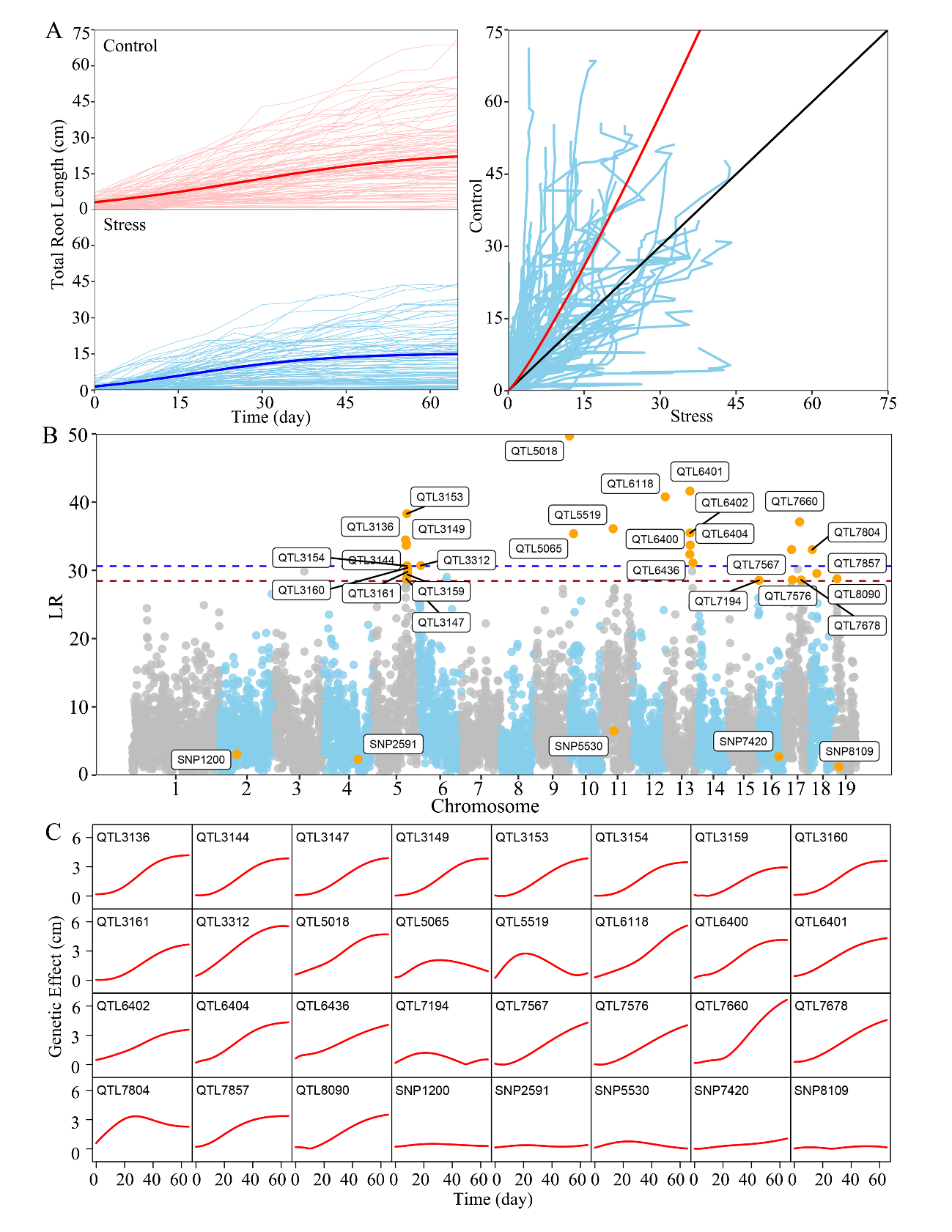


**Figure S1** Composite functional mapping (coFunMap) to identify QTLs that govern the phenotypic plasticity of root growth trajectory in the mapping population of Euphrates poplar. (**A**) Growth trajectories of the total length of adventitious roots (thin lines) grown in salt-free (control) and salt-exposed tubes (stress) during the early ontogeny of juvenile trees (left panel). The mean curve of the F_1_ progeny is fitted by Jenss and Bayley’s growth equation (1). Root growth curves are compared for the same progeny grown in control and stress conditions (right panel). (**B**) Manhattan plot of log-likelihood ratios (LR) for phenotypic plasticity against different chromosomes estimated by coFunMap. Horizontal lines represent the critical thresholds of testcross markers (red line) and intercross markers (blue line) determined from 1000 permutation tests. Many QTLs detected reside at the genomic region of candidate genes (Table S1). Five insignificant SNPs highlighted at the bottom of the Manhattan plot are chosen for a further analysis from the perspective of genetic networks. (**C**) Genetic effect curves of 27 QTLs, plus the five insignificant SNPs chosen, for the phenotypic plasticity of root growth.


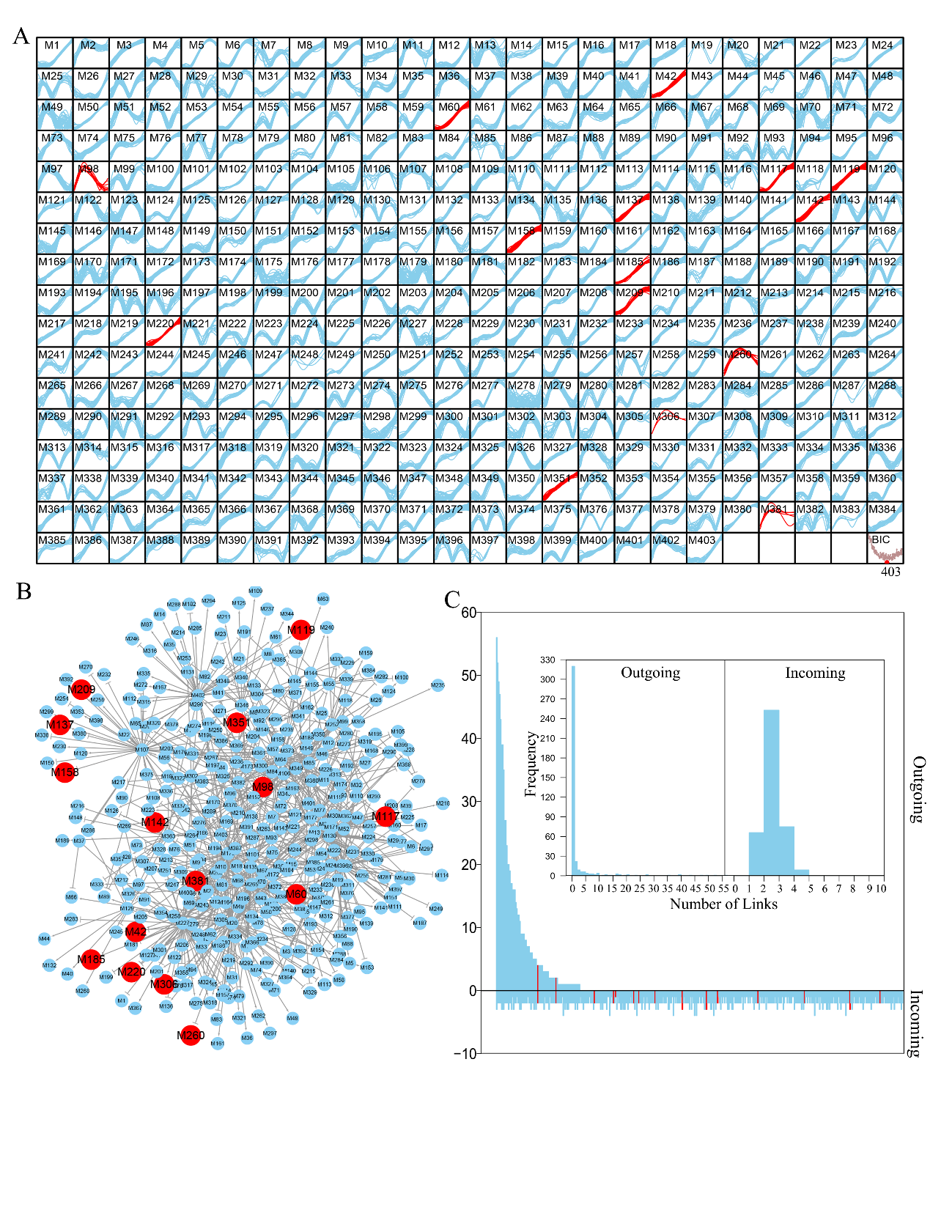


**Figure S2** Identification of network communities from large scale genetic networks for the phenotypic plasticity of root growth trajectory in the mapping population of Euphrates poplar. (**A**) Genetic effect curves of 403 modules detected by functional clustering. BIC analysis shows 403 as an optimal number of modules. (**B**) A 400-node coarse-grained genetic network reconstructed by the mean effect values of each module, where arrowed lines and T-shaped lines stand for activation and inhibition, respectively, with the thickness of lines proportional to the strength of regulation. (**C**) Numbers of outgoing links (upper) and incoming links (lower) are plotted against individual modules, in which the frequency distribution of link number is given. In all figures, modules containing QTLs are highlighted in red.


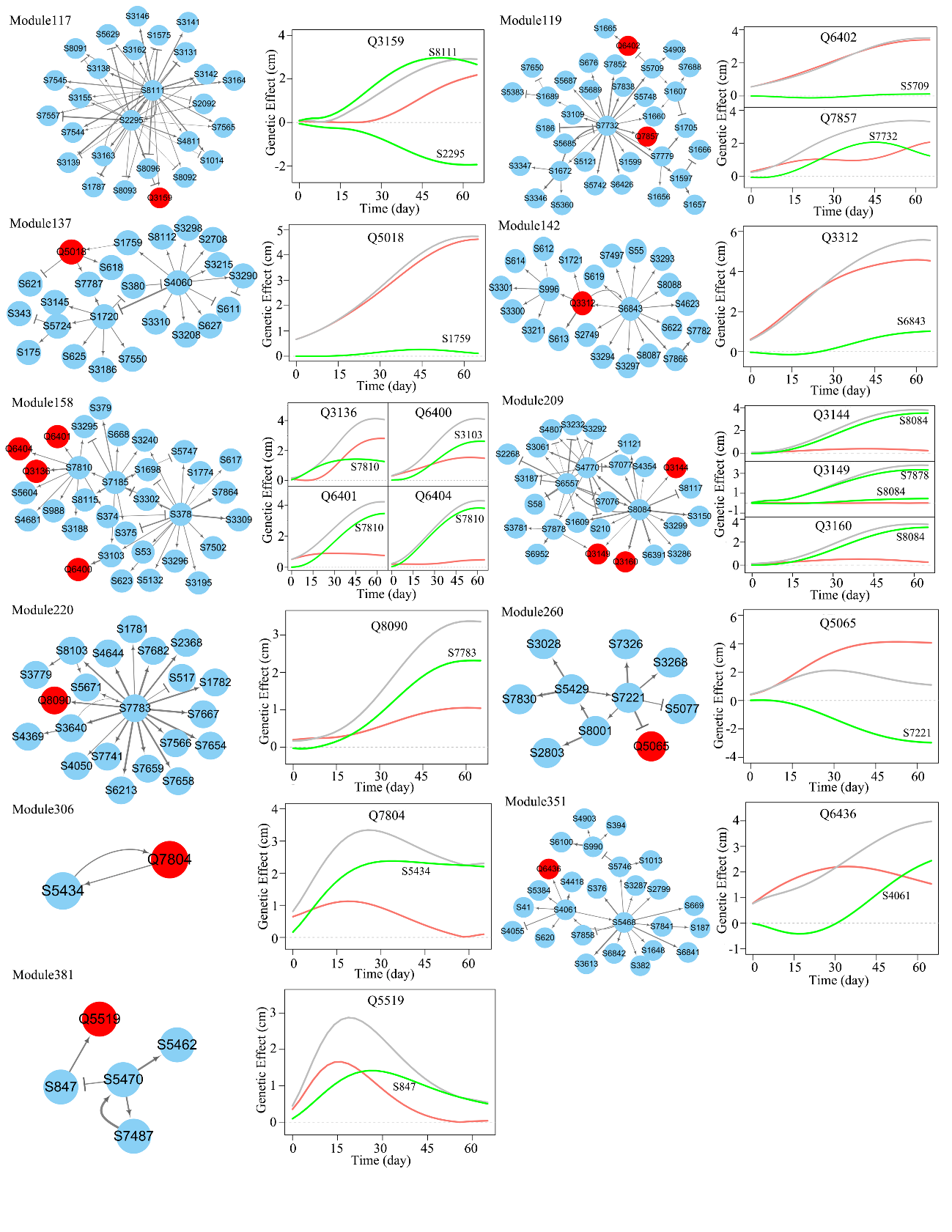


**Figure S3** Fine-grained genetic networks at the SNP level for QTL-containing modules M1170, M119, M137, M142, M158, M209, M220, M260, M306, M351, and M381 underlying the phenotypic plasticity of root growth trajectories in the mapping population of Euphrates poplar. In the networks, arrowed lines and T-shaped lines stand for activation and inhibition, respectively, with the thickness of lines proportional to the strength of regulation. QTLs are highlighted in red. Next to the networks, net genetic effect curves (black line) of each QTL from the above four different modules are decomposed into independent effect curves (red line) and dependent effect curves (green line) due to regulation by other SNPs.


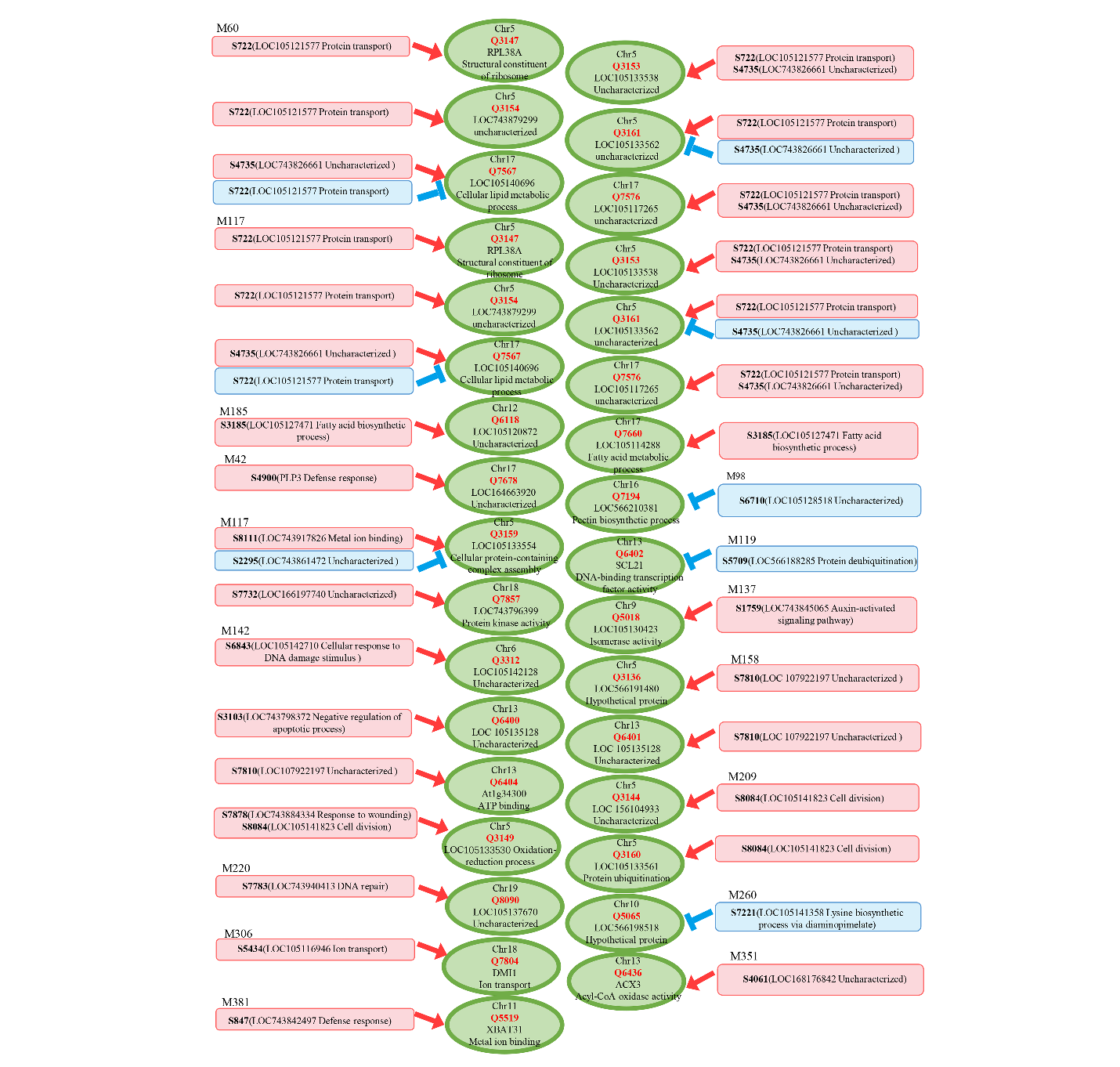


**Figure S4** Gene enrichment analysis of QTLs for the phenotypic plasticity of adventitious root growth trajectory in the mapping population of Euphrates poplar detected by coFunMap and the SNPs that regulate these QTLs. A QTL is up-regulated (red arrows) by SNPs within warm frames and/or down-regulated (blue Y-shape) by SNPs within cold frames. The pattern and strength of regulation for the QTLs by other loci may be an important but neglected driver of salt resistance.


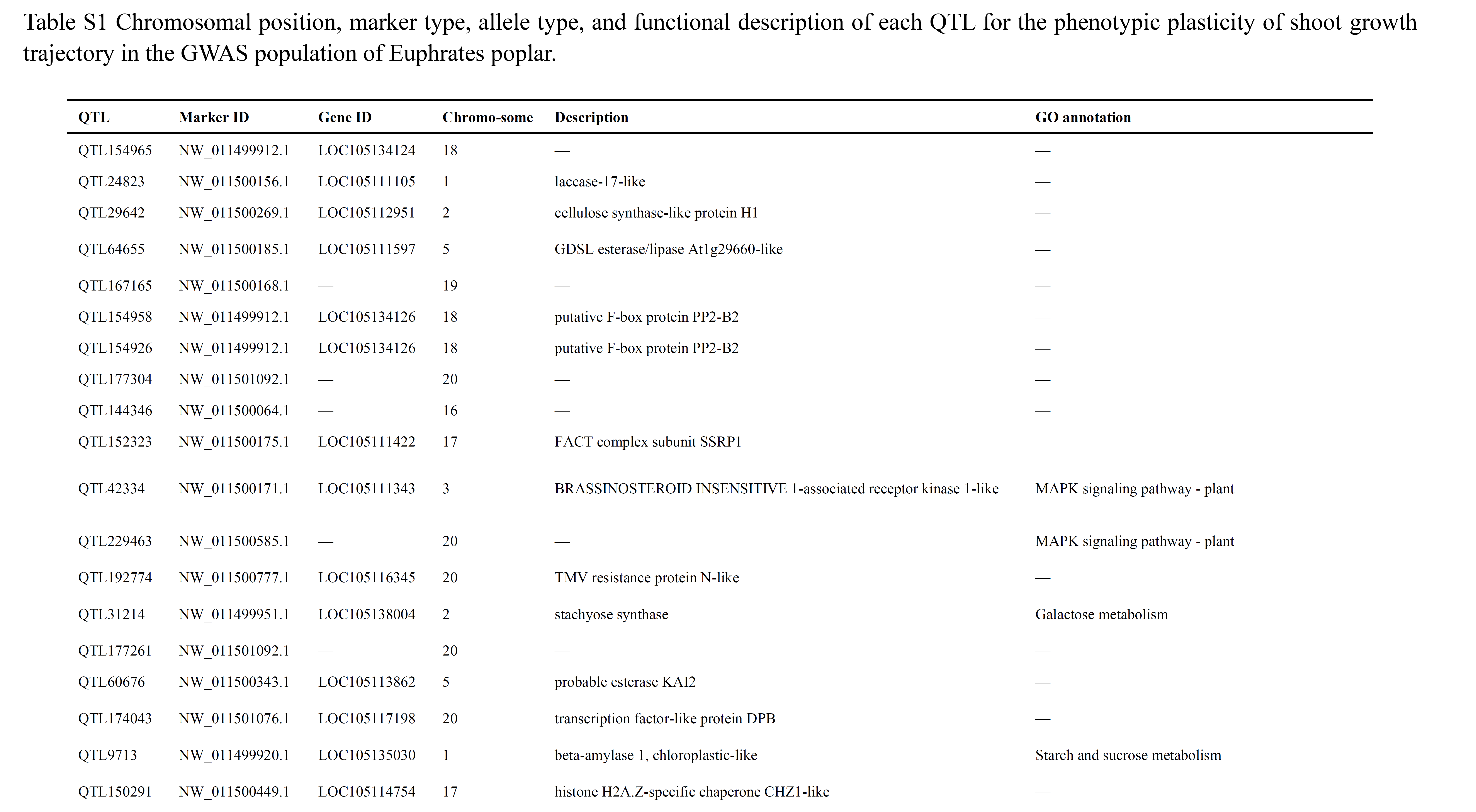


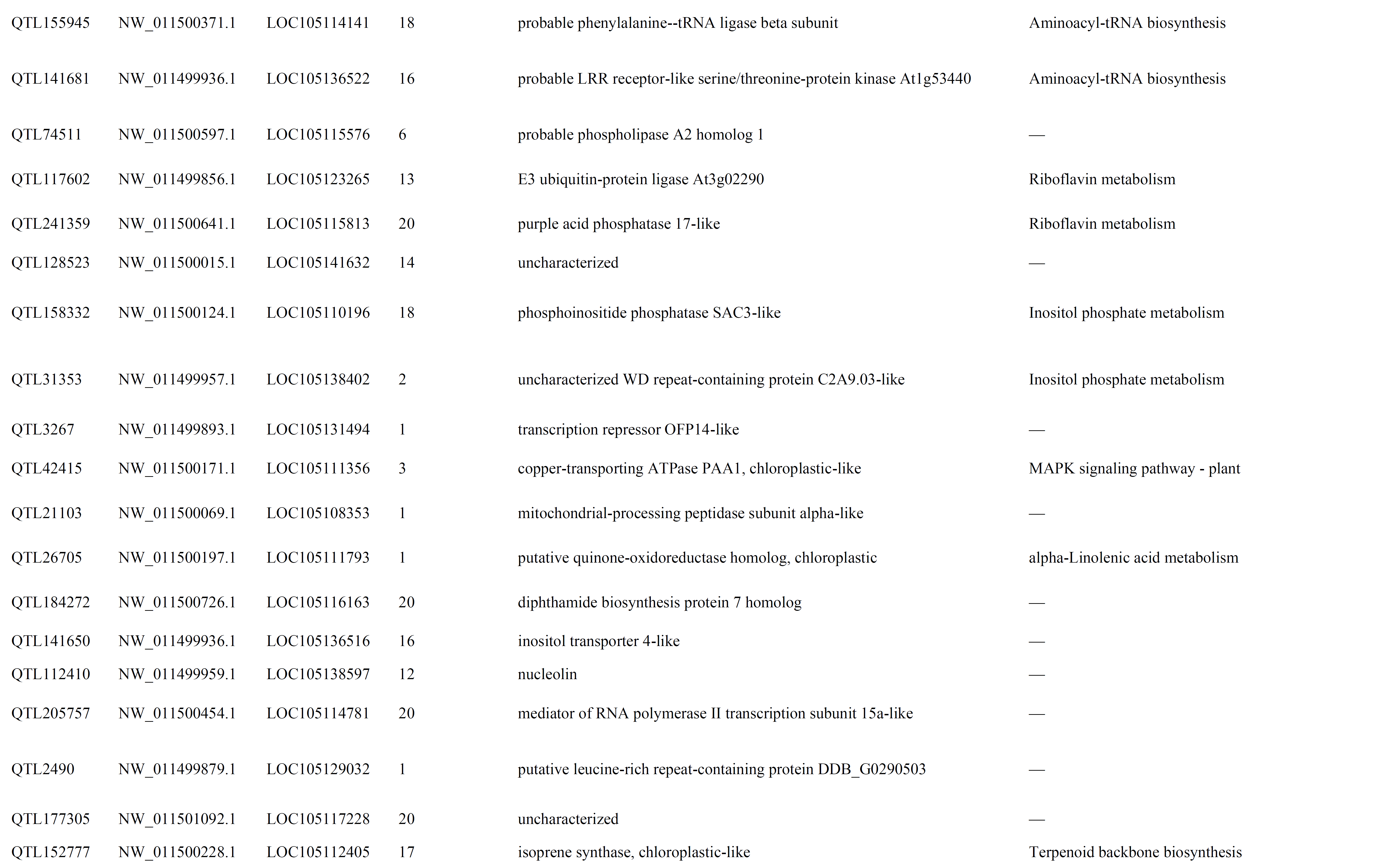


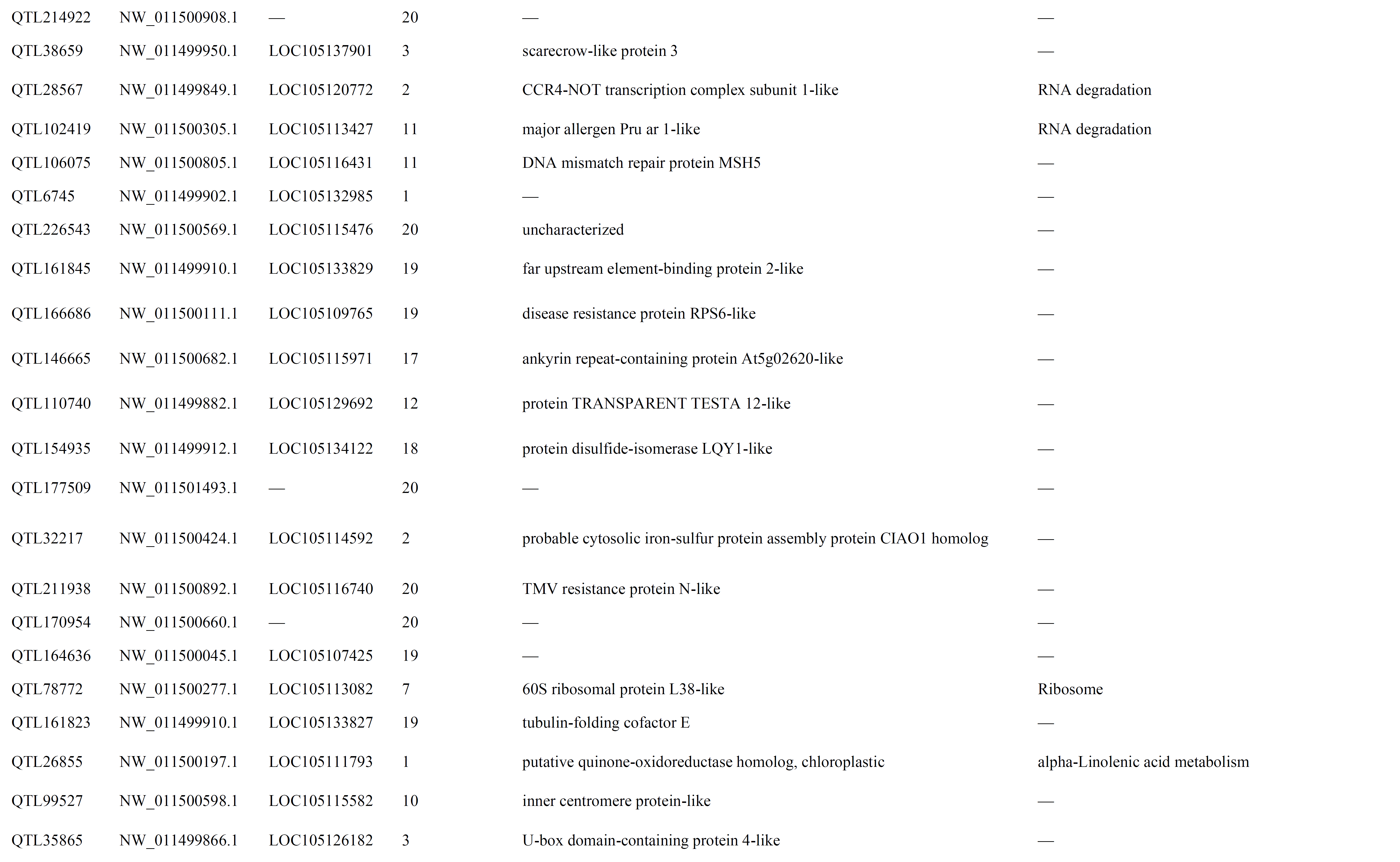


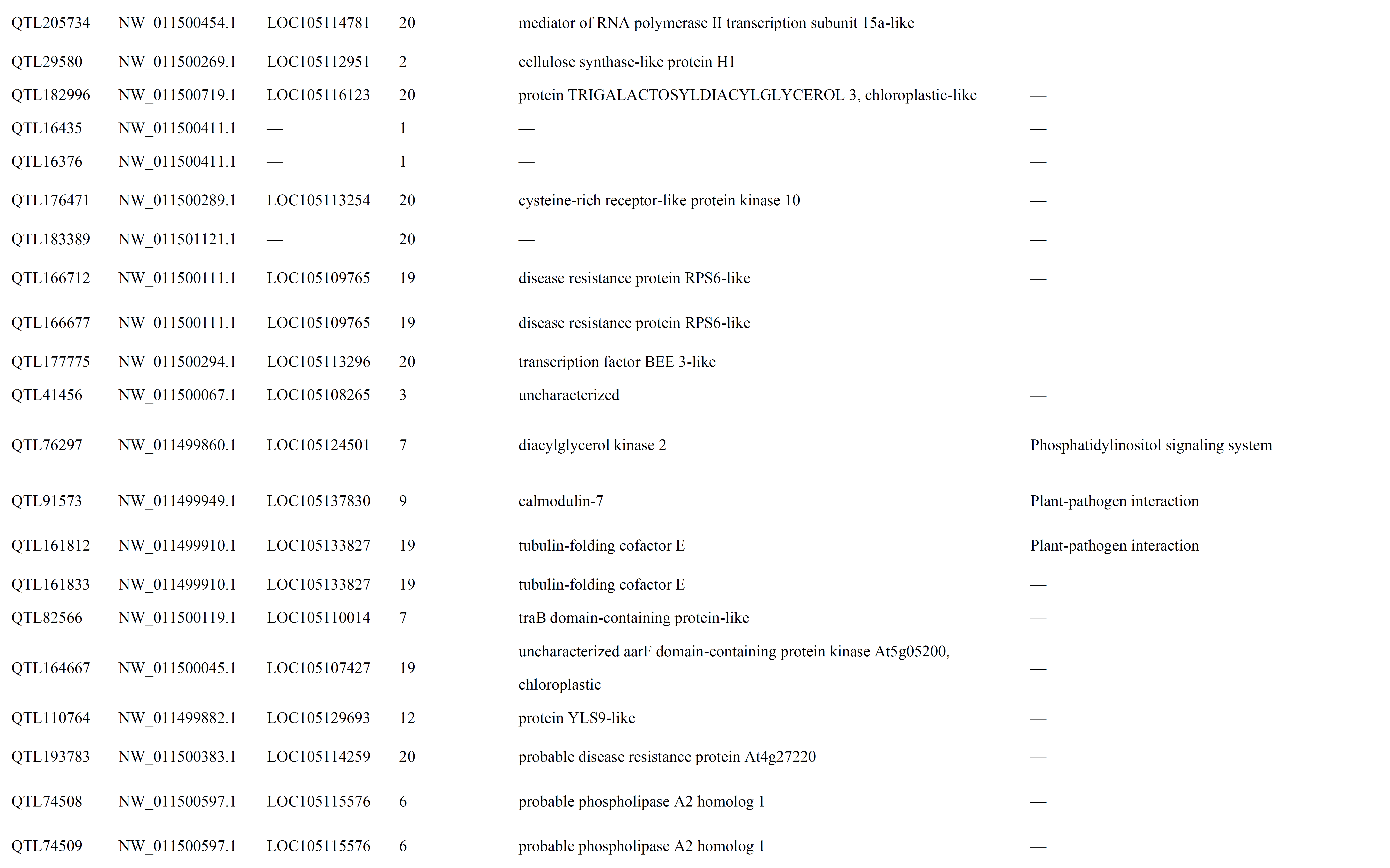


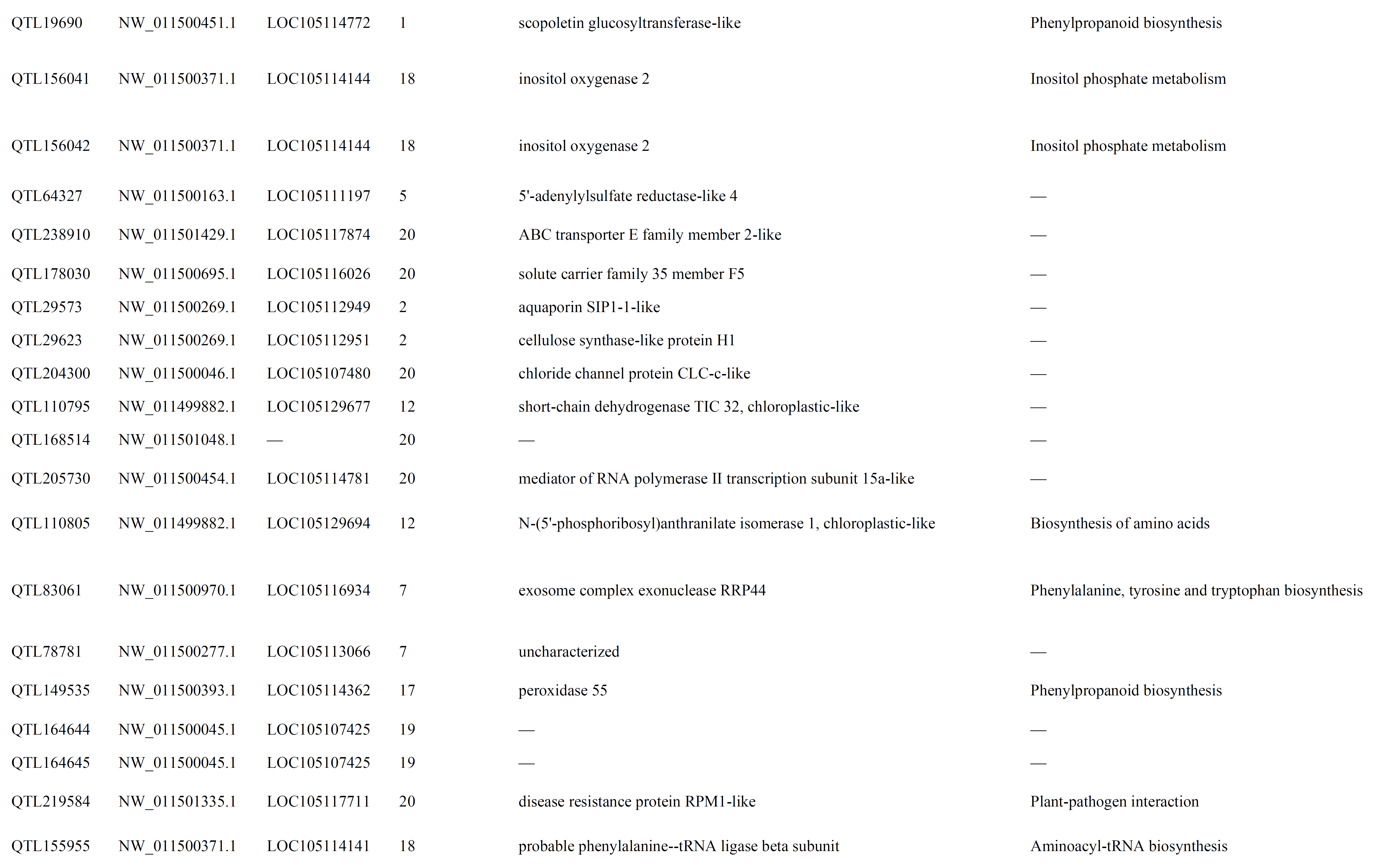


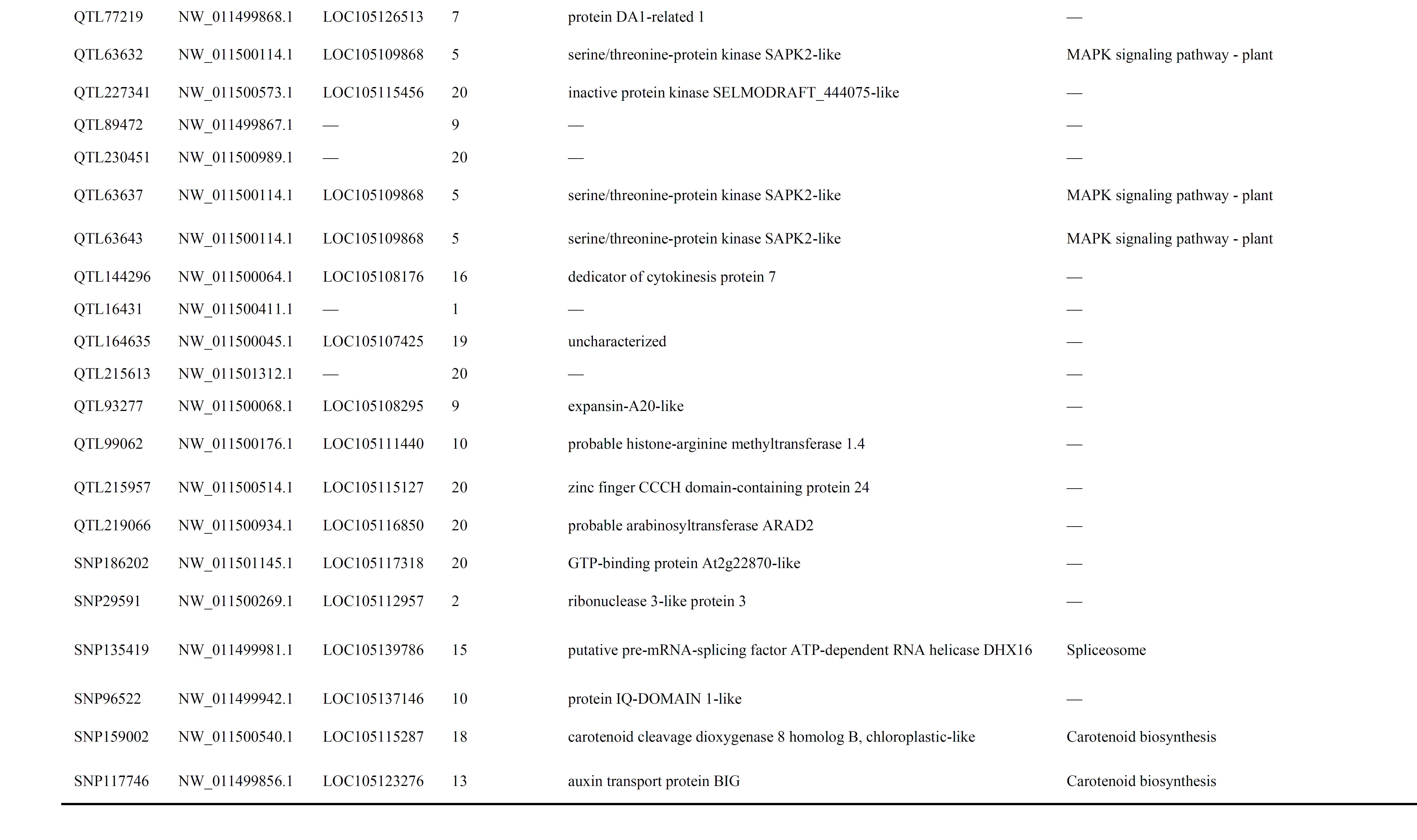


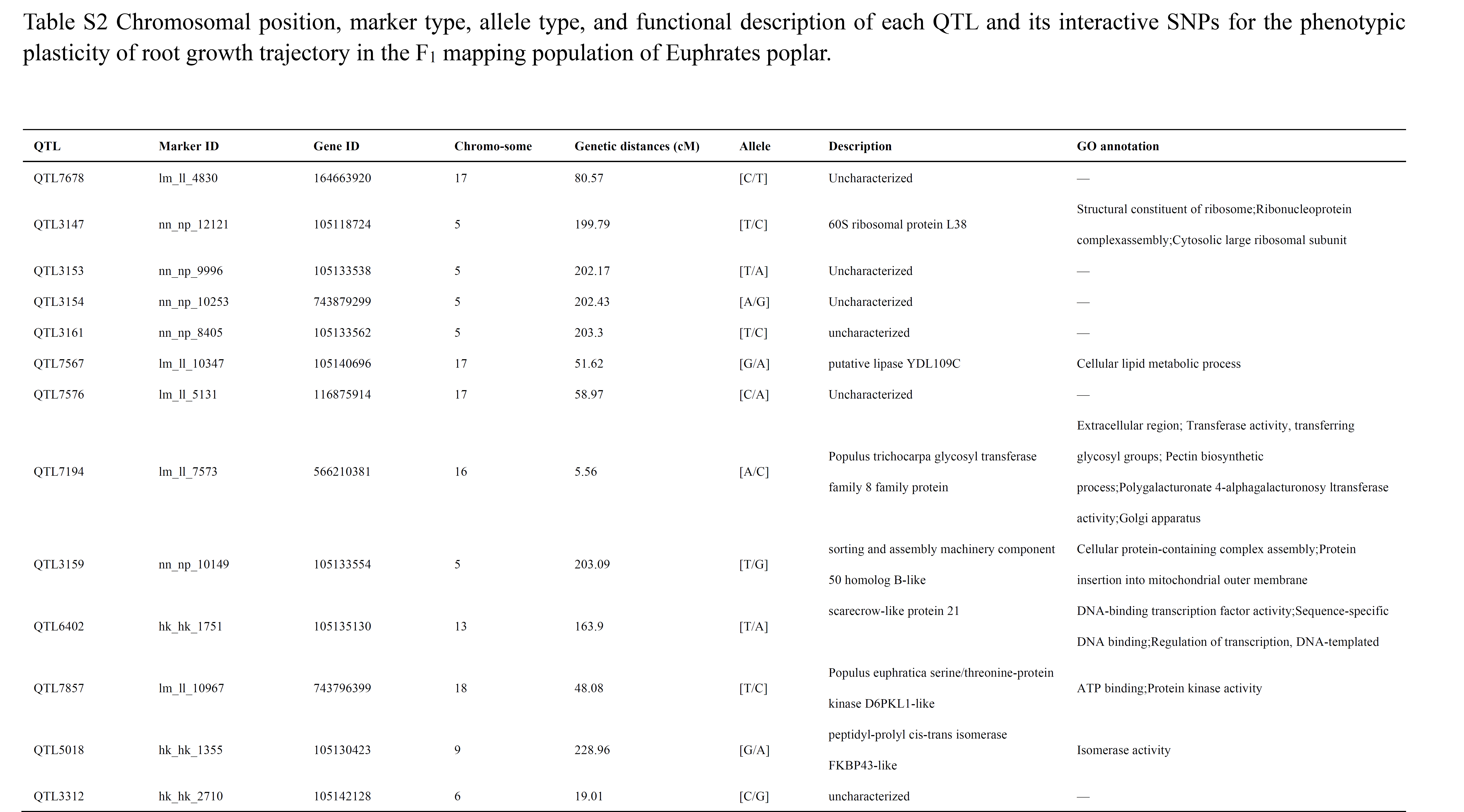


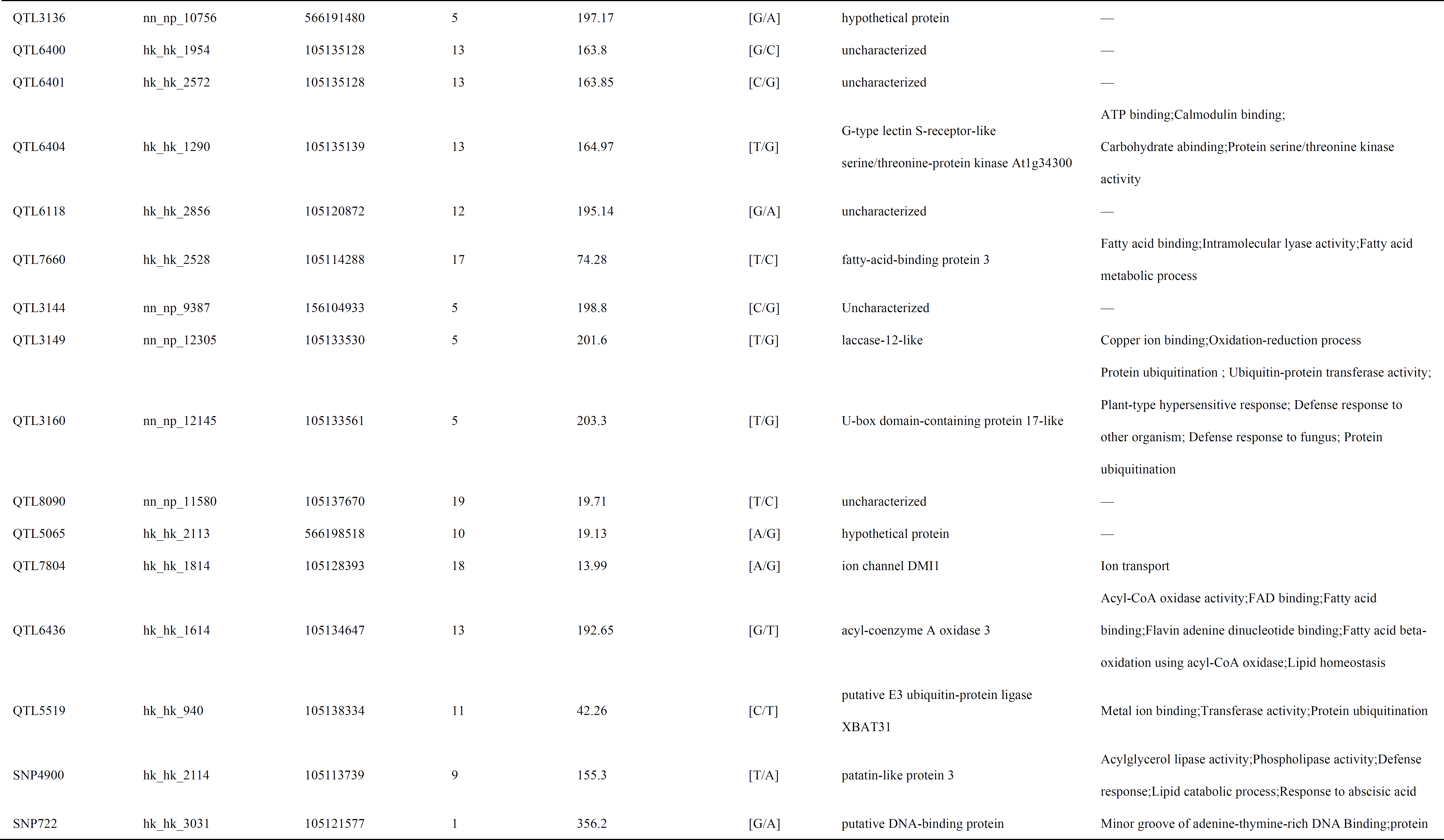


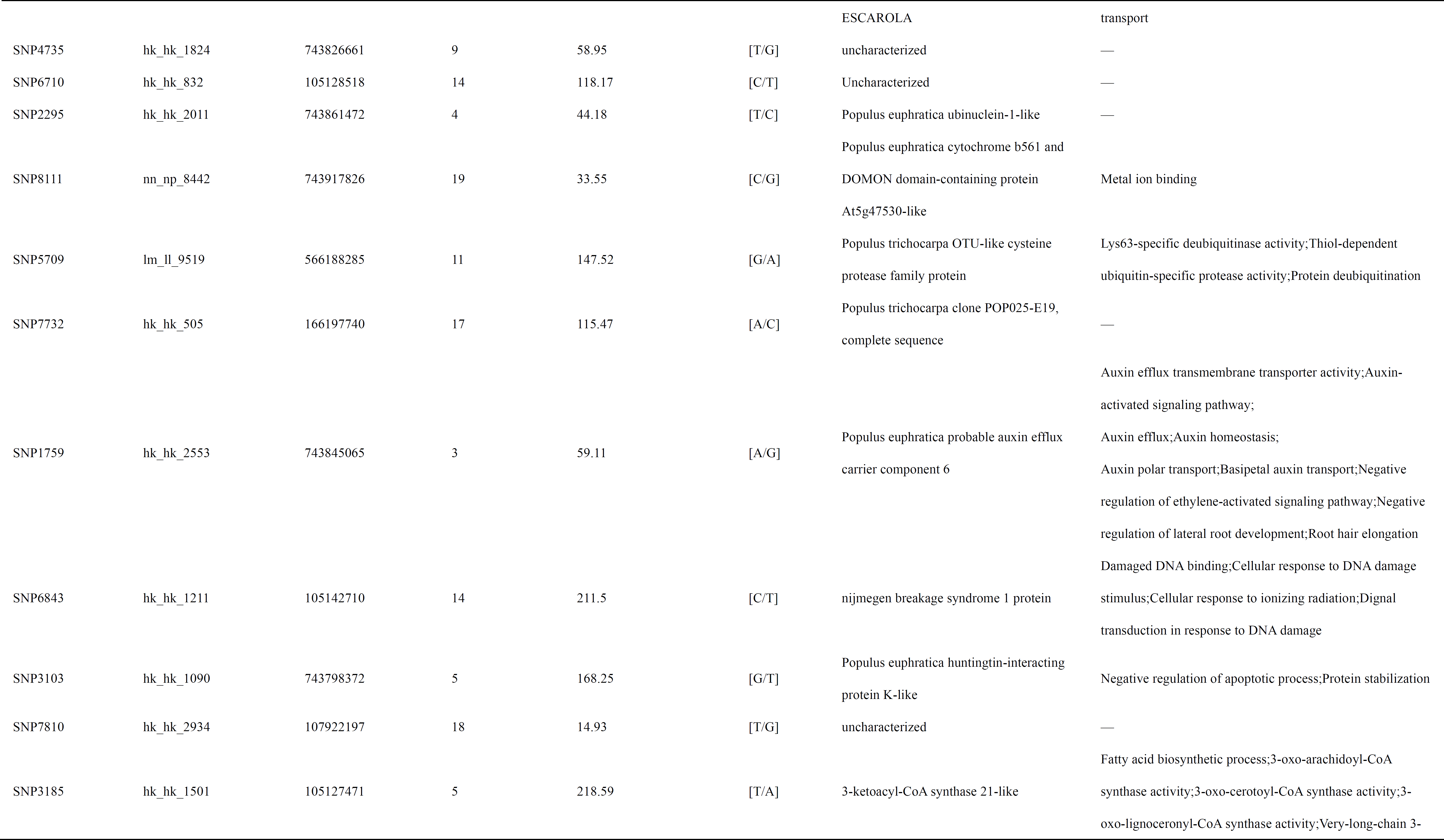


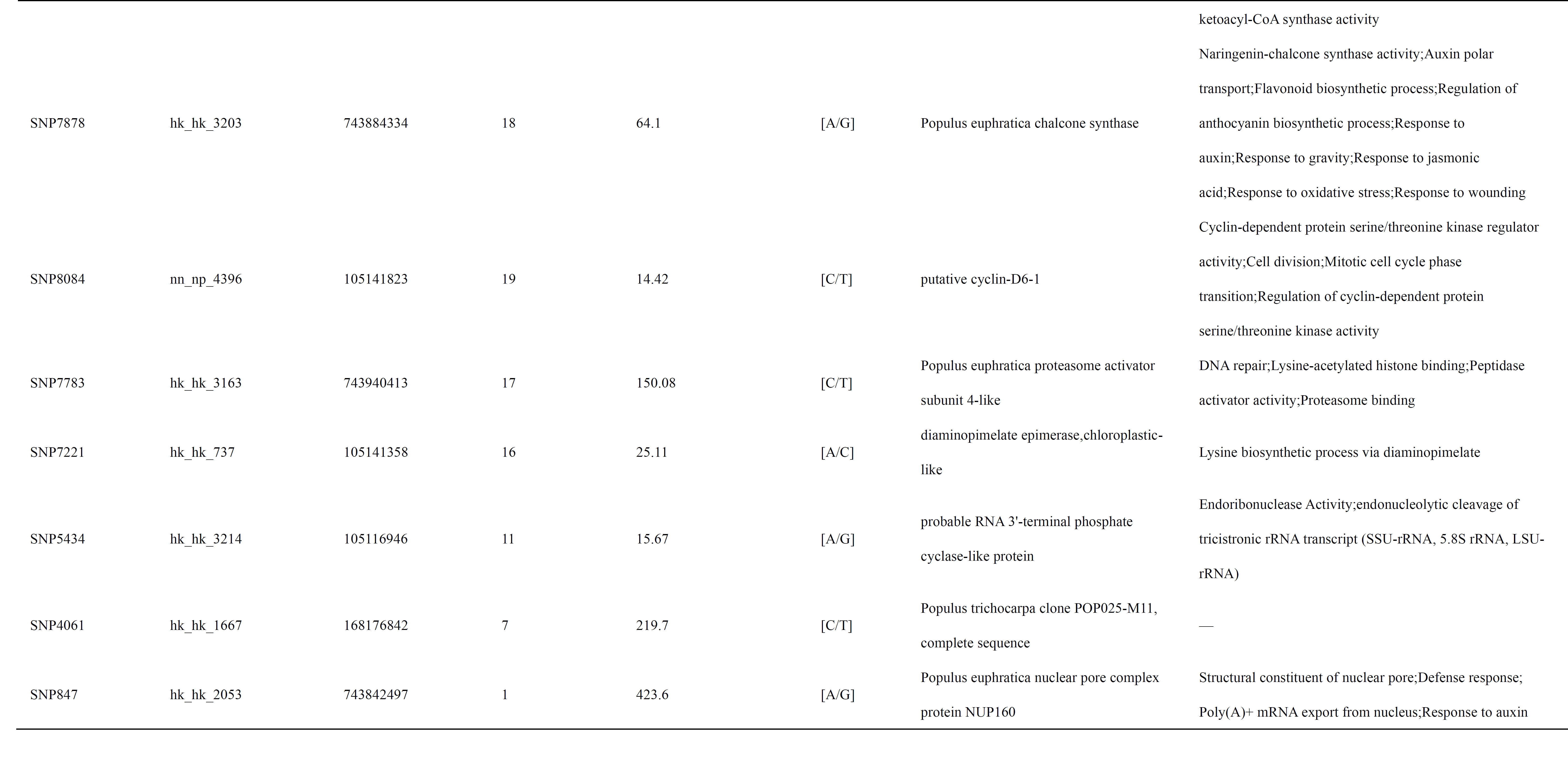

Supplement: Web_Material_uhac135 [file web_material_uhac135.docx]
